# Automatic Detection of Moths (Lepidoptera) with a Funnel Trap Prototype

**DOI:** 10.3390/insects14040381

**Published:** 2023-04-13

**Authors:** Norbert Flórián, Júlia Katalin Jósvai, Zsolt Tóth, Veronika Gergócs, László Sipőcz, Miklós Tóth, Miklós Dombos

**Affiliations:** 1 Institute for Soil Sciences, Centre for Agricultural Research, ELKH, Herman Ottó út 15, H-1022 Budapest, Hungary; 2 Plant Protection Institute, Centre for Agricultural Research, ELKH, Pf. 102, H-1525 Budapest, Hungary

**Keywords:** automatic counting system, pest detection, pheromone trap, remote sensing, real-time monitoring

## Abstract

**Simple Summary:**

Modern pest control is based on correct timing protection and the avoidance of unnecessary insecticide use. Therefore, we must know the exact time of pest gradation and activity. Using automatic insect traps allows insect activity detection without considerable human intervention. The proper use of automatic catching, counting, and data forwarding in the field has not been fully resolved yet. This study presents a modified trap prototype used for automatically catching and counting flying insects, mostly pest moths, in the field. Here, we present the modifications to the construction of our trap design. During the pilot field tests, the new probe prototypes provided real-time, time-series data sets for each of the six pest moth species monitored. Environmental noise was reduced and filtered out. Detected data were forwarded to a web interface where end-users could further process or download the data. With this new device, moths’ daily and seasonal flight patterns could be followed and described. This knowledge may provide an opportunity for more precise forecasts of population outbreaks.

**Abstract:**

Monitoring insect populations is essential to optimise pest control with the correct protection timing and the avoidance of unnecessary insecticide use. Modern real-time monitoring practices use automatic insect traps, which are expected to be able to estimate the population sizes of pest animals with high species specificity. There are many solutions to overcome this challenge; however, there are only a few data that consider their accuracy under field conditions. This study presents an opto-electronic device prototype (ZooLog VARL) developed by us. A pilot field study evaluated the precision and accuracy of the data filtering using an artificial neural network(ANN) and the detection accuracy of the new probes. The prototype comprises a funnel trap, sensor-ring, and data communication system. The main modification of the trap was a blow-off device that prevented the escape of flying insects from the funnel. These new prototypes were tested in the field during the summer and autumn of 2018, detecting the daily and monthly flight of six moth species (*Agrotis segetum*, *Autographa gamma*, *Helicoverpa armigera*, *Cameraria ohridella*, *Grapholita funebrana*, *Grapholita molesta*). The accuracy of ANN was always higher than 60%. In the case of species with larger body sizes, it reached 90%. The detection accuracy ranged from 84% to 92% on average. These probes detected the real-time catches of the moth species. Therefore, weekly and daily patterns of moth flight activity periods could be compared and displayed for the different species. This device solved the problem of multiple counting and gained a high detection accuracy in target species cases. ZooLog VARL probes provide the real-time, time-series data sets of each monitored pest species. Further evaluation of the catching efficiency of the probes is needed. However, the prototype allows us to follow and model pest dynamics and may make more precise forecasts of population outbreaks.

## 1. Introduction

Monitoring insect populations is essential both in the field of ecology and practical pest management. It is a constitutive part of Integrated Pest Management (IPM): the primary approach for decreasing environmental loads in agriculture [1]. The most widespread monitoring tools in IPM are insect traps, which help to detect and estimate the pest population in a given plantation and set action threshold levels. However, maintaining traps during the season requires high human and financial efforts due to frequent inspections, counting, and the identification of trapped insects. Furthermore, species identification requires high-level taxonomic expertise, which is increasingly lacking nowadays. Additionally, there is a delay in data communication due to expert sample processing. Parallel counting in extended areas is challenging with human work [2]. These disadvantages of insect traps may hinder correct and timely decision-making [2].

New real-time monitoring technologies provide key information as to where, when, and to what extent the emergence of pests is expected, thus allowing reasonable timing for the necessary protection and omission of unnecessary insecticide treatments. The precise timing of the different protection methods will become increasingly crucial as selective insecticides become widespread. We experienced rapid development over the last decade in insect detection technologies. Automatic counting in IPM-related research was mainly obtained by traps installed with different types of sensors [3,4,5].

Population size estimation, real-time use, and species specificity are the three most important and expected features of automatic insect detection. Liu et al. [5] distinguished two main types of sensing: acoustic and machine vision systems (MVS), which were used for different insect groups. In recent decades, considerable efforts have been made to specify and improve these two lines of methods for flying insects such as moth species.

Acoustic traps (microphones) were used either outside the trap for the detection and identification mostly of mosquito species [6] or inside the McPhail or Jackson traps and were based on the species-specific spectrums or frequencies of wingbeats [7,8]. In the second research direction, among the MVS methods, the most common ones were optoelectronic devices: laser beam, infrared beam, video and photo tools, CCD sensors, or a combination of them.

For the pest moth forecast, automatized sticky and pheromone traps have been developed with detection systems. Currently, the most widespread insect detection method is the use of different image analysis software [2]. Most of these automatic traps catch the insects and detect them either dead or stunned [9,10]. Sticky traps equipped with cameras can send pictures directly to the laboratory, where image analysis software, with the help of artificial intelligence and neural networks, can gain a high level of species recognition (e.g., [9,11,12]). These systems are easy to use and have a high potential due to their rapid development of artificial intelligence; however, they have some disadvantages. The sticky surface of the traps is easily overloaded and needs relatively frequent human intervention, especially in the case of mass swarming. The probes (traps equipped with sensors) also need a continuous power supply. In addition, the position of the insects as they are caught may make species recognition challenging [3].

Regarding flying insects, photo shootings are hardly possible because of their rapid movement. For this reason, traps equipped with optoelectronic sensors detect specimens when they are falling or flying through the sensor field [13,14]. These sensors detect the light differences when the incoming insects interrupt the infrared (IR) beam. Probes with IR sensors have lower energy consumption. Some solutions use reflected light instead of emitted light [15]. These detectors recognise colour and morphological patterns and gain a higher level of species specificity. Species specificity can be traditionally achieved by using sex pheromones as bait [16], which can also be joined to electronic probes, creating another line and gaining species specificity at automatic traps [17]. Funnel traps with pheromones collect insects continuously without the problem of overloading. Sex pheromones attract male individuals for moth species, so population and activity parameters can be estimated only based on male specimens.

Despite the widespread literature of these studies and the fast development of devices, only a small fraction of the above-mentioned methods have been applied under field or semi-field conditions (e.g., greenhouses and storage halls). Several constraints hinder or delay the use of these methods under field conditions, such as high environmental noise, high energy consumption, and oversaturation. Further details about existing probes and sensors detecting flying insects (primarily moths and fruit flies) in the field are summarised in Supplement S1.

In recent years, we have developed optoelectronic sensors [18,19] to detect small arthropods living in the soil. The infrared (IR) sensor ring was further developed to detect invertebrates of different body sizes [20]. By using this IR sensor ring, we constructed automatic traps (probes) to detect small invertebrates living in the soil [21] and the adults of the Western Corn Rootworm (*Diabrotica virgifera virgifera*; Coleoptera: Chrysomelidae) [22]. Finally, to detect flying insects, we used the same IR sensor ring installed in the CSALOMON^®^ VARL funnel traps produced by the Plant Protection Institute, Centre of Agricultural Research, ELKH, Budapest. In this present study, we describe the construction of this new device. We also present the precision and accuracy of the new probe by comparing the number of detected and caught insects. Pilot study results are also shown to demonstrate the advantages of the new probe.

## 2. Materials and Methods

### 2.1. Description of the New Probe (ZooLog VARL)

The new probe prototype called ZooLog VARL has been developed to catch flying insects such as moths. It automatically counts and also forwards the data to end users. The equipment comprises three main parts: a trap, a sensor, and a data communication system.

#### 2.1.1. Modified VARL Trap

**Figure 1:**
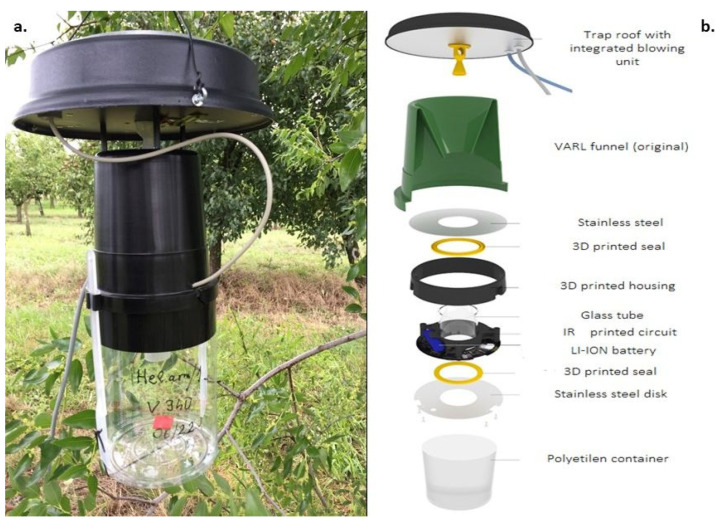
ZooLog VARL probe prototype in the field (**a**) and its 3D schematic diagram (**b**). The probe was built to detect flying insects, mostly pest moths and other lepidopteran pests. The probe was baited with a pheromone lure of the target insect; insects dropping through the funnels into the catch container could not escape. An infrared sensor-ring detects the individuals, and a blow-off system directs the flying insects toward the catch container. For further details, see Supplement S2.

The base of the ZooLogVARL is the commercially available VARL funnel trap belonging to the CSALOMON^®^ pheromone trap family (Plant Protection Institute CAR, ELKH, Budapest, Hungary, www.csalomoncsapdak.hu, (accessed on 23 February 2023) [23,24]). When flying individuals enter the funnel (top opening outer diameter: 13 cm, funnel hole diameter: 3 cm, height of funnel: 16 cm), they drop through the hole at the bottom of the funnel and cannot escape (Figure 1). Insects are attracted by the species-specific sex pheromone bait (for males) placed under the roof of the trap (20 cm diameter). While flying through the funnel of the ZooLogVARL, insects were detected by an infrared (IR) sensor ring, which was built right under the lower hole of the funnel (Figure 1). Initially, the funnel was transparent or green in VARL traps, but we changed it to black to prevent sunlight from getting into the trap body, which could cause false detections in the IR sensor. A second funnel was built under the IR sensor ring to decrease the probability of escape for insects already caught and detected.

In the case of insects, which actively fly into the probes, as they fly around and through the sensor, their movement can severely multiply the number of detections. According to our observations in the field, this “in and out” movement is more frequent than expected. First, to solve the problem of multiple counts, we used a double row of IR sensors, which count with fly-in and fly-out [13,25,26]. However, this double-layered sensor ring solution did not work appropriately in our case because the two layers of the sensor-ring could not be set far enough from each other to identify the direction of the flights. If we set the two sensor ring far enough, we could create an appropriately long tube that the insects did not enter or fly through. Holguin et al. [14] used kill strips at the trap entrance. That way, moths fell through the sensor field, but they were not killed and caused miscounting by the sensors and low accuracy. To resolve the problem of multiple counting, we developed a blow-off system, which activated after detection and blew down the insects. In previous testing of the ZooLogVARL, several methods were tried (without the active movement of insects, with top and down ventilator, and compressed air pressure, see Supplement S2). The finalised blow-off device was built above the funnel, under a plastic lid, to prevent escaping individuals from proceeding backward from the funnel, which could decrease counting precision (Supplement S3). When the IR sensor-ring detects an individual, the blow-off system is automatically turned on three times for one second. It blows off the insect into the sample container placed under the funnel. This transparent plastic sample container was made longer in size than the original one (20 cm high, 13.5 cm diameter) to allow the appropriate functioning of the blow-off system. To increase the dark area in the sample container, a 4.5 cm wide black tape was also put around the upper part of the plastic container (Figure 1a). According to our field observation, if the sensor detected a moth, no escape was detected due to the blow-off mechanism.

#### 2.1.2. Sensor-Ring

The construction of the sensor ring is fully described in Balla et al. [20]; therefore, only a brief description is provided here. The optoelectronic sensor was placed around the glass tube. If an insect interrupted the path between the receiver and the emitter, the optoelectronic sensor recorded the event. Then, it activated the blow-off system, which blew off the flying insect. The used IR sensor ring (IRSR-1) [20] had a wide sensor field, thereby detecting arthropods of considerable size, for example, Noctuid moths. The sensor was placed in a waterproof plastic tube with rubber rings. It is connected to the probe with a stainless-steel disk (Supplement S2). In previous work [20], we tested the detection accuracy of the sensor ring with dead animals. For moths with a size of 6.35–23.94 cm, such as *Ephestia kuehniella* (Phycitidae), *Operophtera brumata* (Geometridae), and *Autographa gamma* (Noctuidae), detection accuracy was revealed to be 100%.

#### 2.1.3. Data Communication System

As has already been described by Tóth et al. [22], the ZooLog system was designed for the online monitoring of arthropods. It works with its own data forwarding system, a central database, and a Web interface. Due to the solar panel power supply, the probe detects insects in real time and sends data daily throughout the season. A logger is connected to each probe that transmits the sensor data via the Internet. The results of the detection data can be downloaded from or managed directly on a ZooLog Online Web Interface.

The materials used to produce the trap and the electronics cost about 300 euros. The total production cost of the trap prototype was 650 euros. The blow-off system accounted for half of the costs.

### 2.2. Field Tests

**Figure 2:**
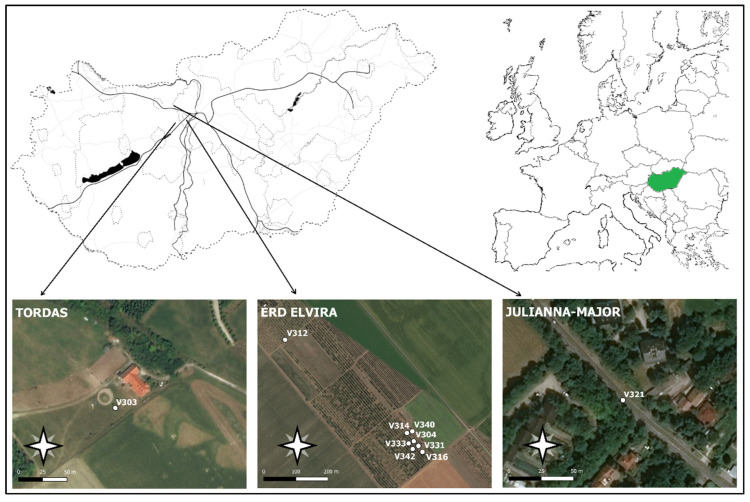
Map of the experimental orchards. White dots indicate the locations of the ZooLog VARL probe prototypes.

Automatic monitoring was conducted in three locations in Hungary (Figure 2, Table 1). Six moth species (*Agrotis segetum*, *Autographa gamma*, *Helicoverpa armigera*–Noctuidae; *Cameraria ohridella*–Gracillariidae, *Grapholita funebrana*, *Grapholita molesta*–Tortricidae) were monitored in the field in 2018 to test the new probe’s automatic counting accuracy and precision (Table 1). Initially, we started the experiment with several probes (depending on the number of species expected in the area). However, due to several problems (steal, mechanical crack), we could use the data for the whole season of 10 probes for six different Lepidopteran species. Eight probes were functioning at Érd-Elvira major (Experiment Station of Research Institute for Fruit Growing and Ornamentals). One probe was used at Julianna-major (Ecological Experimental Station of the Plant Protection Institute, Centre for Agricultural Research, ELKH) and in a private orchard nearby Tordas village (see Table 1).

The probes were in operation from the beginning of June until the middle of October 2018, depending on the species activity (Table 2). Commercially available pheromone lures (CSALOMON^®^ traps, Plant Protection Institute CAR, Budapest, Hungary) for the investigated moth species were used in the probes. Baits were changed for a fresh one every six weeks, as proposed by Tóth [33]. Our goal was to test the accuracy of the probes for different species and to not follow the gradation patterns. Therefore, we missed the spring peaks of moth gradation in 2018.

The number of moths caught was recorded automatically in real-time, and data were sent to the server daily. The probes were also manually checked daily, and the number of captured specimens was recorded. The number of catches and the corresponding detection data could be retrieved and compared through an inbuilt query.

### 2.3. Data Analysis

#### 2.3.1. Data Filtering

From the raw dataset produced by the IR sensor ring, false detections were filtered out according to the same method used by Tóth et al. [22]. The IR sensor ring produces intensity data from the eight sensors surrounding the tube at the bottom of the capturing funnel. The pattern of the eight figures was used to filter out the noise data induced by other non-target species or plant leaves. We confirmed IR signals to be good or false detections for the filtering procedure based on manual checking in short periods. These verified data were used in the deep-learning analysis. Deep-learning data analysis was performed with TensorFlow [34] on Github [35]. We used 19,228 data in the learning database for 12 species (containing four other species not involved in this analysis, such as *Agriotes* species and *Diabrotica c. virgifera*). The learning database contained the eight IR intensity figures and the manual decisions on whether it was a true or false detection. The script was written in TensorFlow using the framework Keras in Python. It is available here:

https://colab.research.google.com/drive/1Nzngi_4UxipvoQt5O0Ez4AciGTcZNiCh#scrollTo=vVa1Q4Qwoef5&line=5&uniqifier=1 (accessed on 21 February 2023).

#### 2.3.2. Statistical Analysis

All statistical analyses were performed in R [36]. The daily data of the ten probes were analysed separately for each species.

An artificial neural network (ANN) approach was used to estimate the performance of the data filtering procedure with the standard evaluation metrics (accuracy, precision, recall, and F1 score). According to the contingency matrix of binary classification, the accuracy is the rate of all true values and the total number of detections. Precision means the rate of true positives and all positive values. The recall is the rate of true positives and the sum of true positives and false negatives. F1 score is defined as the harmonic mean of precision and recall.

$$A=\frac{TP+TN}{TP+FP+TN+FN}\;P=\frac{TP}{TP+FP}\;R=\frac{TP}{TP+FN},$$

where *A*: accuracy, *TP*: true positive, *TN*: true negative, *FP*: false positive, *FN*: false negative, *P*: precision, *R*: recall.

The accuracy and reliability of the sensor system were calculated by comparing automatic (and filtered) and manual counts using linear models. For under- and overestimation, the intercept and the slope of the model were tested against 0 and 1, respectively (‘multcomp’ package, [37]). All data were ln (x + 1) transformed prior to the analysis to meet normality assumptions.

Based on the data derived from the automatic detection, some examples were presented to visualise the target species’ local daily and temporal activity. Local polynomial regression fitting (loess) was applied for smoothing time-series data (ggplot2 [38]), lubridate [39] packages).

## 3. Results

### 3.1. Data Filtering Using ANN (Artificial Neural Network) Approach

False detections caused by light effects, plant leaves carried by the wind, or non-target insects were filtered out. In Table 2, we show the statistics of the contingency tables of the species detections. The filtering accuracy of ANN was always higher than 60%; in the case of species with larger body sizes, it reached 90%.

### 3.2. Detection Accuracy and Performance of the New Probe

**Figure 3:**
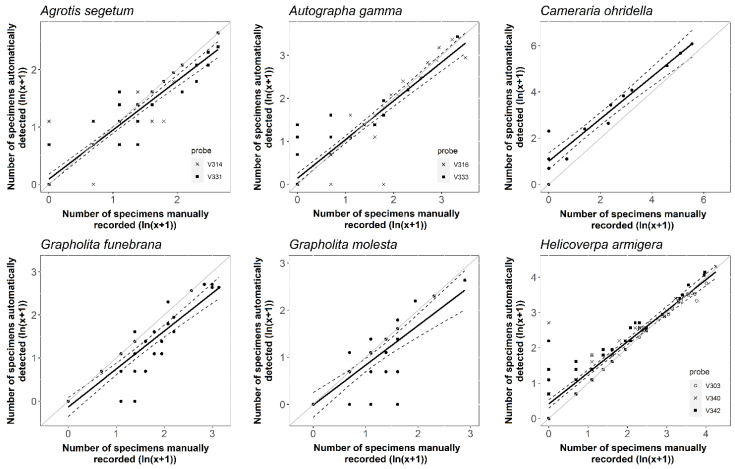
The relationship between the number of detected and captured individuals for six different Lepidopteran species. The solid lines show the predicted values from the linear model, and the dashed lines show a 95% prediction interval. The solid grey lines indicate the equality between the two variables.

The comparison of detected and caught, manually counted data revealed that detection accuracy varied by species (Table 3, Figure 3). The accuracy values for the six target species ranged from 84 to 92% on average (Table 3). The probe was the most accurate for *C. ohridella* species (91.55% on average, see Table 3), while it provided the lowest average accuracy for *G. molesta* (84.29%). Except for the latter species, ZooLog VARL probes achieved a relatively high level of reliability (R^2^, Table 3). The slope of the linear regression lines was significantly lower than one for all the target species. Moreover, the intercepts of the models significantly differed from 0 in the case of *H. armigera*, *A. gamma*, and *C. ohridella*. However, the intercept was higher than one only for the *C. ohridella* species showing a significant overestimation.

### 3.3. The Temporal and Daily Activity Patterns of Target Species

**Figure 4:**
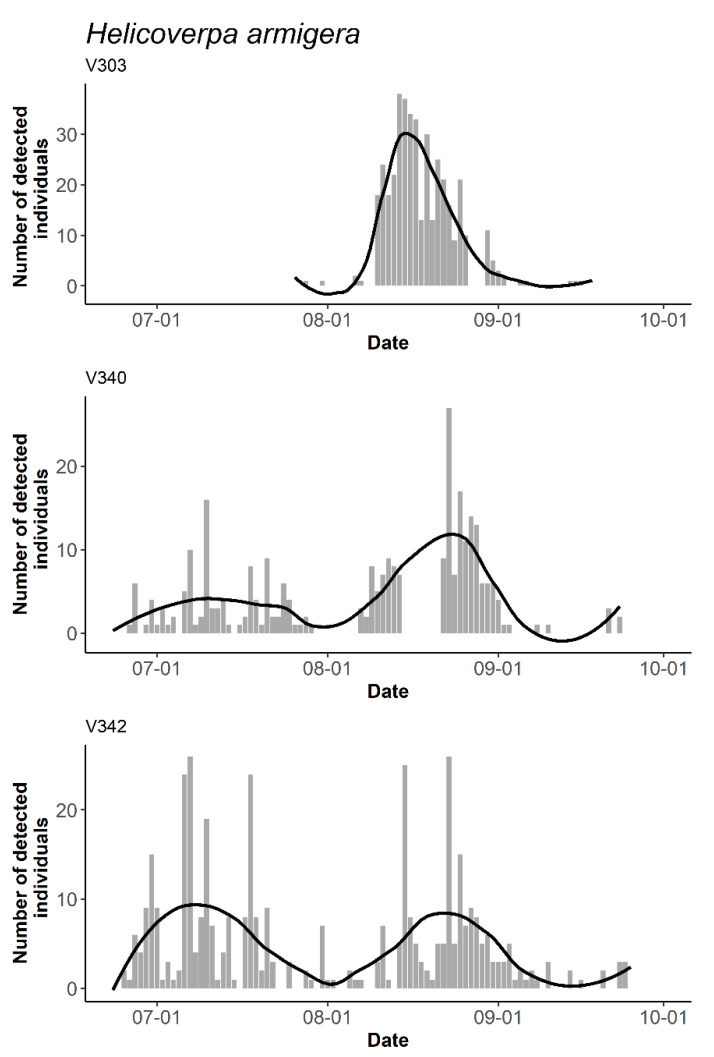
An example of the automatic detection of individuals for target species over time during the monitoring period. The number of detected *Helicoverpa armigera* individuals from three different probes. Local polynomial regression fitting (loess) was applied for smoothing time-series data.

With ZooLog VARL probes, the seasonal activity of the six Lepidopteran species was followed during the study period (for example, see Figure 4 for *Helicoverpa armigera*, the patterns of other species are shown in Supplement S4). The differences between the taxa was experienced by the number of individuals caught. *Cameraria ohridella* was the most abundant, while, for example, *Agrotis segetum* or the two *Grapholita* species were represented by a lower number of specimens (Supplement S4).

The individuals of *A. segetum* were detected in higher numbers in late June and July, while the specimens of *A. gamma* were most active in late June and early September, even if the latter was not supported by both trials (Supplement S4). The higher flight activity of *H. armigera* was recorded in early July and mid-August. In contrast to the other two trials, the third one (V303) started later and took place at another location (Tordas site), resulting in a slightly different pattern (Figure 4). After the initial peak in July, *G. molesta* increased its temporal activity. The individuals of *C. ohridella* and *G. funebrana* flew the most at the beginning of the study period, in late June, and then they were present in decreasing numbers in the traps (Supplement S4).

**Figure 5:**
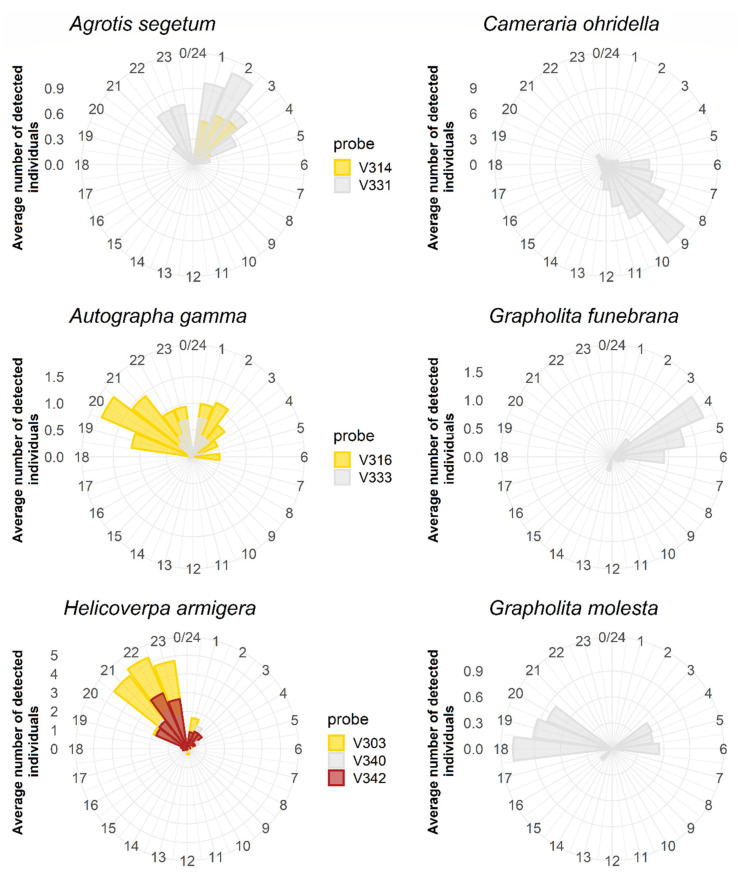
Daily flight activity periods of the six target species. The data were generated by the ZooLog VARL probes for at least 11 weeks from July to October 2018, depending on the flight period of the species. Different colours represent data deriving from different probes.

ZooLog VARL probes also enabled the daily activity of target species to be followed (Figure 5). Typical nocturnal species were *A. segetum* (mainly between 10:00 p.m. and 3:00 a.m.), *A. gamma* (mostly between 7:00 p.m. and 3:00 a.m.), and *H. armigera* (mainly between 8:00 and 11:00 p.m.). By contrast, the individuals of *C. ohridella* were exclusively observed between 6:00 and 12:00 a.m. Different daily patterns characterised the two *Grapholita* species. *Grapholita funebrana* was active only at early dawn (between 4:00 and 6:00 a.m.). At the same time, *G. molesta* was found in the early morning period (see explanation in the Discussion) and flew in the late afternoon and evening (between 6:00 and 8:00 p.m.).

## 4. Discussion

The ZooLog VARL probe was prototyped for detecting and automatically counting the flying insects, especially pest moths. The probe was based on a commercially available CSALOMON^®^ VARL funnel trap. Species specificity was achieved with the sex pheromones widely used to attract insect pests. With this, the catch of non-target species was minimalised. The new trap used active trapping with an inbuilt blow-off device. With that, fly-in and fly-out movement were prevented, which might cause the double counting of insects at sensing. Although a statistical comparison of the catching efficiency of the new probe prototypes used the blow-off system, the classic CSALOMON funnel traps were still missing, we did not experience a significant decrease in the number of catches (personal communication of Miklós Tóth).

The current technology in automatic traps works with pheromones and usually uses sticky sheets where insects are stuck (see Supplement S1). The identification of target species is based on pictures. The only drawback of these methods is their possible oversaturation. Our probes have a lower chance of oversaturation.

Considering the sensors under laboratory conditions, precision was proved to be 100% for the inspected moth species [20]. However, the precision obtained in the field had lower values due to environmental noise. This detection was based on the disruption of the IR beam, of which the drawback was the high environmental noise. False detections can be derived from the wind, solar radiation, and other interventions, especially when the traps hang on the trees. Looking at the raw detection data, we could conclude that the environmental noise for IR sensors was high. Filtering out noise data was a critical part. The eight adjacent IR sensors in the sensor ring provided eight intensitive figures when an object crossed the sensor field. We used the pattern of these eight values next to each other to filter the noise. The more adjacent sensors were activated, and bigger-sized insects could have been detected.

We used an ANN machine-learning procedure to characterise the targeted species, i.e., true detections. This filtering was more efficient for bigger species than small species since the light-induced detection patterns or dust particles gave similarly smaller patterns. This way, however, we could filter a large number of light noises that could be seen in the number of true negatives (TN). False negatives (FN) might come from the non-target species we could not filter, while false positives (FP) were lost during filtering. FP was very high at *Cameraria ohridella*. However, this deep-learning method allowed us to filter out a considerable number of false detections that frequently occurred as IR sensors. Without this or another filtering method, this device would be unusable.

We tested the detection accuracies by comparing the number of target species to the number of filtered detections. In the field tests, we achieved relatively high average accuracies (84.29–91.55%), depending on the species with a 6–40 mm size (Table 3). In the IPM practice, the number of pest moths was informative, as a rough rule of thumb, between 0–3, 3–10, and >10. The biases of the detected number of individuals varied between 8 and 12% on the logarithmic scale (see Table 3, Slopes). Therefore, we can assume that the device could be used for pest management. VARL probes achieved a relatively high level of reliability, except in the case of *Grapholita molesta*, where R^2^ was 0.614. This corresponds with the results of Preti et al. [40], who investigated stuck insects (*C. pomonella*) with a camera trap. They explain it with the misidentification of non-target species. Although the recognition mechanisms used are different, this could be an explanation in our case, where the *G. molesta* pheromone can attract the males of other species as well.

The data communication system (own forwarding system, central database, Web interface) allows end users to obtain daily, real-time, filtered data. The loggers were set to send data once daily to save energy effectively. Analysis reports can be automatically performed and downloaded.

In another electronic trap using the same IR sensor ring, we could gain a 60–70% detection accuracy under semi-field conditions for soil arthropods with sizes of 0.5–2.5 mm [21]. We gained a 95.84% detection accuracy in agricultural use for the larger-sized western corn rootworm (4.4–6.8 mm) under field conditions [22].

We developed our probes for remote field use. Therefore, the energy consumption of the devices should be low. The sensors work continuously, however, on only a slight consumption. A detection event activates the system with higher energy consumption. This function saves energy, so our probes can work with a single solar panel for months. However, the use of solar panels can sometimes be challenging in dense orchards or forests.

Despite the occasional lower reliability, the probe can give an alert sign of swarming events. The new probes can follow the exact time of pest occurrence and dynamics. We obtained a more detailed picture of the seasonal dynamics of the six different species investigated. During the study period, we found the different seasonal activities of the different moth species, which corresponded to the flight patterns known from the literature. Although the probes caught different quantities of specimens and we did not follow the entire flying season of the species, their seasonal flight activity could appropriately drown up. *Agrotis segetum*, *Autographa gamma*, *Helicoverpa armigera*, and *Grapholita molesta* species were characterised by a bimodal activity pattern. Despite the low number of probes used, these results were consistent with the previous results from the literature [41,42]. *H. armigera* and *G. molesta* have three peaks in their activity [43], but we missed the first ones due to the late start of the investigations. The number of *Cameraria ohridella* and *Grapholita funebrana* species displayed a decreasing trend with an initial peak at the end of June. However, we may have also missed the first peaks.

Our knowledge about male moths’ exact daily flight periods is scarce [44]. This aspect of the species’ circadian rhythm can also be studied with ZooLog VARL probes. Previously, the circadian rhythm data of species were obtained by hourly manual checks of the traps. With ZooLog VARL probes, it can be more accurate. The activity time of typical nocturnal species, such as *A. segetum*, *A. gamma,* and *H. armigera*, was well detected with the new probes. *C. ohridella* was exclusively observed between 6:00 and 12:00 a.m. Different daily patterns characterised the two *Grapholita* species. According to our data, *G. funebrana* was active only at early dawn (between 4:00 and 6:00 a.m.). By contrast, *G. molesta* flew mainly in the late afternoon and evening (between 6:00 and 8:00 p.m.). These two species share some of their main pheromone components. In the case of the *G. funebrana* lure an inhibitory component for *G. molesta* was added. Therefore, the trap baited with *G. funebrana* lure caught only this species [45]. However, for *G. funebrana*, the selective attractant inhibitor is not known [46]. Therefore, traps baited with *G. molesta* pheromone attracted *G. funebrana* as well [47]. The daily flight periods of these species are different: *G. funebrana* flies at dawn, while *G. molesta* in the late afternoon and early evening [17,48]. Moth catches in the *G. molesta* lures was also detected in the early morning. However, these detections were probably *G. funebrana* catches, the species of which are very similar to *G. molesta* by morphology. For this reason, in the case of *G. molesta*, to filter out *G. funebrana* catches, a morning filter could readily be applied in the future, thus presenting the very first selective *G. molesta* trap in the long history of pheromone trap applications.

It has to be stated that these results are for demonstration and are based on the data of only 1-1 (and a maximum of three) probes for different species. More probes at different locations would allow the observation of more accurate patterns which could be compared with the environmental data.

Automatic detection is a crucial element of IPM. With that, the estimation of the location and the exact time of actual pest outbreaks would be more precise and reliable. As species phenology shifts due to recent climate change, automatised traps could become more essential for the determination of new patterns. The time of spraying can be optimised. By reducing unnecessary sprayings, more environmentally friendly farming and a decrease in plant protection costs could be achieved. Mating disruption, primarily based on sex pheromones, is a non-insecticide method, which could be more effective with the knowledge of the exact sexual activity and (flight) time of insects [49].

Automatisation involves less human effort as well. Human field intervention would be necessary only for refreshing the pheromone lure and emptying the catching container. The number of trap inspections could be decreased, and counting specimens, which is the most time-consuming work, could also be performed automatically [2]. In extensive farms or remote areas, fuel costs and human effort can further be reduced [50]. Compared to the manual checks of probes, the ZooLog VARL probe would provide a cost-effective method for automatic pest detection.

Before commercialisation, several aspects have to be solved yet. The price of the traps needs to be decreased during commercial production, but theft and physical damage protection are two of the essential aspects that must be solved. Ascolese et al. [51] suggested that the position of the traps, due to the solar panel supply, could affect the catching efficiency. The use of probes in dense vegetation has to be solved as well. Solar panel cables can be extended; that way, probes could be used in denser vegetation, moving solar panels up to the canopy or to the edges of the vegetation.

ZooLog VARL probes can be used to detect any species for which funnel traps (VARL) are recommended while the pheromone lures work properly. Due to pheromone contamination, one probe can detect only one species. The sensors are already used in different trap types, such as soil traps and KLP traps for *Diabrotica virgifera* [22]. That way, by inserting sensors into different traps or places, there is a high potential for sensing different types of insects, where species specificity can be solved. For example, it is possible to insert sensors into the entrance of honeybee hives to measure the activity of these pollinators. Alternatively, it is possible to measure aero fauna as well, where we are interested in the density of insects. The design and data communication system could also allow environmental sensors’ attachment and proper function. As Preti, Verheggen, and Angeli [2] suggest, insects’ population dynamics can be better modelled with these innovations.

With automatic traps, real-time data can be gained from different locations simultaneously. In addition to actual farming use, our device can also be helpful for ecological, entomological, and agro-ecological studies. Data could be gained with high precision for the exact activity time of insect species supported by data on environmental conditions. With that in mind, population outbreaks could be better modelled, and more precise forecasts could be made.

## 5. Conclusions

During the field test, ZooLog VARL probe prototypes provided real-time, time-series data sets for each of the six pest moth species monitored. High environmental noise, as a common problem during field application of automatic probes, was filtered out. The other frequent problem when counting flying insects is their fly-in and fly-out movement which can cause severe overestimations. This issue was solved with the new blow-off device, which prevented escape from the trap. These probes can work with a high detection accuracy without considerable human intervention. With the use of sex pheromones, high species-specificity can be achieved. Through a data communication system, the results of the detection data are automatically forwarded to a web interface where data can be further processed or downloaded. With this new device, time and costs could be saved for end users in the future. In the case of insect monitoring, the new device provides a more accurate estimation of their daily and seasonal activity. Here, in these preliminary experiments, we focus on the precision and accuracy of detection in this field. However, as in the case of any new trap solution, the catching efficiency of the probe should be evaluated in the field by comparing the catches of the prototype to classical funnel traps. This is essential since the catching efficiency influences the settings of the action thresholds in IPM.

## Figures and Tables

**Table 1:** Field tests on different species.

Species	Host and Symptoms	Flight Period	Daily Rhythm	Wingspan/Body Length (mm)	Test Site	Test Period	Number of Probes
*Agrotis segetum* Den. and Schiff., 1775 (turnip moth)	polyphagous, larvae feeding on the part of the host plant at the soil level or above 1–2 cm	2 generations/year 1—beginning of May to the middle of June 2—beginning of July to September	4–5 h to the scotophase [27]	30–40/15–20	Érd-Elvira major orchard	2018.06.06.–2018.10.15.	2
*Autographa gamma* L., 1785 (silver Y)	polyphagous, larvae feeding on the leaves	2 generations/year 1—beginning of May to the middle of June 2—middle of June to the beginning of October	during the scotophase [28]	35–40/15–20	Érd-Elvira major orchard	2018.06.22.–2018.10.15.	2
*Cameraria ohridella* Deschka and Dimič, 1986 (horse-chestnut leafminer)	horse-chestnut (*Aesculus hippocastanum*), larvae bore mines into the leaves	3 generations/year 1—end of April to the beginning of May 2—in June 3—middle of July to the beginning of September	beginning of photophase, lasts for 4–5 h (7 p.m.–2 p.m. in July in Hungary) [29]	6–8/5	Julianna-major, row of horse chestnut trees	2018.06.25.–2018.10.15.	1
*Grapholita funebrana* Treitschke, 1985 (plum moth)	*Prunus* spp. (plum, apricot) Larvae bore inside the fruit feed on the flesh around the seeds	3 generations/year 1—middle of April to the middle of May 2—end of May to the end of June 3—beginning of July to the beginning of August	end of the scotophase to the beginning of photophase [30]	9–13/6–9	Érd- Elvira major orchard	2018.06.21.–2018.10.15.	1
*Grapholita molesta* Busck, 1916 (oriental fruit moth)	peach, almond, apricot, medlar, pear, apple spring–larvae bore into fresh shoots summer–larvae bore into ripening fruits	3 generations/year 1—middle of April to the middle of May 2—end of May to the end of June 3—beginning of July to the beginning of August	during the scotophase [31]	9–13/5–7	Érd-Elvira major orchard	2018.06.21.–2018.10.15.	1
*Helicoverpa armigera* Hübner, 1808 (cotton bollworm)	polyphagous, larvae feed on the generative parts of the plant	3 generations/year 1—end of May to the beginning of June 2—middle of July to the middle of August 3—end of August to September	during the scotophase [32]	30–40/12–20	Érd-Elvira major orchard	2018.06.22.–2018.10.15.	2
					Tordas orchard	2018.07.25.–2018.10.15.	1

**Table 2:** The performance summary of ANN data filtering procedures. TP: true positive, TN: true negative, FP: false positive, FN: false negative.

Target Species	TP	TN	FP	FN	Filtering Accuracy	Precision	Recall	F1 Score
*Agrotis segetum*	130	1132	63	28	0.93	0.67	0.82	0.74
*Autographa gamma*	201	1113	74	22	0.93	0.73	0.90	0.81
*Cameraria ohridella*	597	242	487	64	0.60	0.55	0.90	0.68
*Grapholita funebrana*	72	158	65	53	0.66	0.53	0.58	0.55
*Grapholita molesta*	78	205	6	20	0.92	0.93	0.80	0.86
*Helicoverpa armigera*	868	1055	246	23	0.88	0.78	0.97	0.87

**Table 3:** The average detection accuracy and reliability (R^2^) of ZooLog VARL probes for the six target species with the slopes and intercepts of linear models fitted to the manually and automatically counted data. Significant *p* values (*p* < 0.05) are highlighted in bold.

Target Species	Number of Measurement Periods	Average Accuracy	R^2^	Slope		Estimation	
				y	*p*	intercept	*p*
*Agrotis segetum*	94	85.64%	0.853	0.86 ± 0.04	**<0.001**	0.09 ± 0.04	0.072
*Autographa gamma*	80	89.94%	0.848	0.90 ± 0.04	**<0.001**	0.14 ± 0.06	**0.030**
*Cameraria ohridella*	16	91.55%	0.932	0.92 ± 0.06	**<0.001**	1.00 ± 0.17	**<0.001**
*Grapholita funebrana*	38	88.02%	0.837	0.88 ± 0.06	**<0.001**	−0.14 ± 0.11	0.449
*Grapholita molesta*	40	84.29%	0.614	0.84 ± 0.11	**<0.001**	−0.01 ± 0.13	1
*Helicoverpa armigera*	111	88.05%	0.887	0.88 ± 0.03	**<0.001**	0.41 ± 0.06	**<0.001**

## Data Availability

The data presented in this study are available on request from the corresponding author.

## Supplementary Materials

The following are available online at https://www.mdpi.com/article/10.3390/insects14040381/s1, Supplement S1: Summary of probes (sensors installed into traps) used to automatically detect flying insects in the field [52,53,54,55,56,57,58,59,60,61,62,63,64,65,66,67,68,69], Supplement S2: Technological description of the ZooLog VARL trap, Supplement S3: Initial phases of the development of the ZooLog VARL probe, Supplement S4: Automatic detection of individuals for the five target species over time during the monitoring period.
