# Supplementary material for: Automatic Detection of Moths (Lepidoptera) with a Funnel Trap Prototype
Source: Insects. 2023 Apr 13;14(4):381. doi: 10.3390/insects14040381 (PMC10145081; doi:10.3390/insects14040381)
Supplement: Supplementary file 1 [file insects-14-00381-s001.zip › Supplement_1.pdf]

Table S1. Summary of probes (sensors installed into traps) used to automatically detect flying insects in the field. Sensor type describes the sensor used in the experiment; Trap type: the used type of trap; Target species; Capturing insect: the method which way they capture the given target taxa; Species selectivity: how do they gain species specificity; Publication

Abbreviations: IR: infrared

Sensor type	Trap type	Target species	Capturing insects	Species selectivity	Publication
**Opto-electronic sensors**					
Double-counting IR sensor	trapping tube placed in a bottle	*Bactrocera dorsalis*	insects cross alive	pheromone	Jiang, et al. [13], Okuyama, et al. [52]
IR interrupter controller, MSP-based sensing device and WSN-based monitoring station, two infrared gates	trapping tube placed in a bottle	*Bactrocera dorsalis*	living insects, (crawling through, the sensor)	pheromone	Jiang, et al. [25], Chuang and Jiang [53]
IR sensors	bucket trap	*Bactrocera dorsalis*, *Cydia pomonella*	insects fall through the sensor	pheromone	Holguin, et al. [14]
double counting IR sensor set	C-DAIS pheromone insect-trapping device	*Spodoptera litura*	insects fall through the sensor	pheromone	Shieh, et al. [26]
IR sensor	cylindrical plastic trap	*Ceratitis capitate*	dead or stunned	pheromone	Goldshtein, et al. [54]

infrared camera	polypropylene food containers with sticky card	*Grapholita molesta*	stuck insects	pheromone or live female	Pérez Aparicio, et al. [44]
IR sensor	cone type trap	*Grapholita molesta*	insects fall through the sensor	pheromone	Kim, et al. [17]
**Laser-radar (lidar) system**					
lidar	no trap	atmospheric insect fauna	no	not, only for quantitative measurement	Brydegaard, et al. [55]
continuous-wave Scheimpflug lidar system	no trap	rice field insects (12 species)	no	not, only for quantitative measurement	Song, et al. [56]
Passive kHz lidar	no trap	aerofauna	free flying insects	for monitoring abundances and fluxes of pests and disease vectors in the atmosphere	Jansson and Brydegaard [57]
lidar	UV light trap	flying insects	free flying insects	for quantifying flying insect abundance; distinguish three insect clusters based on morphology	Kirkeby, et al. [58]
**Acoustic, Opto-acoustic sensors**					
bimodal optoelectronic sensor based on stereo recording device	McPhail trap	fruit flies	insects flying trough	spectrum of the wingbeat	Potamitis, et al. [8]

acoustic piezoelectric counter	modified milk carton trap	*Lymantria dispar*	insects flying in	pheromone	Tobin, et al. [59]
microphone, camcorder	Jackson trap	*Ceratitis capitata*	free flying insects	wingbeat frequency	Mankin, et al. [7]
radio signal	electric insect trap with pheromone, not fluorescent light	*Plutella xylostella*	insects flying into to the trap	pheromone	Hirafuji, et al. [60]
**Audio sensors**					
camera	sticky trap	whitefly, trips and aphid	stuck insects	quantitative measurement	Rustia,et al. [10]
camera	sticky trap	lacewings, diabrotica, whitefly, thrips and aphid	stuck insects	machine vision technique	Solis-Sánchez, et al. [61]
camera	modified McPhail trap	*Bactrocera oleae*	dead insects in the liquid	non specific ammonium sulphate	Doitsidis, et al. [62]
mobile phone camera	sticky pheromone trap (modified Promotrap)	*Cydia pomonella*	stuck insects	pheromone	Guarnieri, et al. [63]
camera	sticky trap	*Aonidiella aurantii*	stuck insects	pheromone	Frewin, et al. [9]
camera	mobile robot car	*Pyralidae*	insects on the vegetation	image analysis (inverse histogram mapping)	Liu, et al. [64]
camera	red coloured Rebell® sticky traps	*Drosophila suzukii*	stuck insects	captive animals, experts + neural network	Roosjen, et al. [12]

digital camera	adhesive liner	*Cydia pomonella*	stuck insects	pheromone	Ding and Taylor [65]
camera	iMETOS iSCOUT® pheromone model	*Grapholita molesta*	stuck insects	pheromone	Ascolese, et al.[51]
camera	PESSL iMETOS iSCOUT® FruitFly model	*Bactrocera dorsalis*	stuck insects	torula yeast	Ascolese, et al.[51]
camera	Trapview Standard model (delta trap)	*Grapholita molesta*	stuck insects	pheromone	Ascolese, et al.[51]
camera	Trapview Self-Cleaning model	*Bactrocera dorsalis*	stuck insects	methyl-eugenol	Ascolese, et al.[51]
camera	Trapview Fly model (cylindrical trap)	*Bactrocera dorsalis*	stuck insects	torula yeast and methyl-eugenol	Ascolese, et al.[51]
camera	sticky sheet	*Resseliella theobaldi*	stuck insect	pheromone	Sipos, et al. [66]
camera	sticky cards	*Drospohila suzukii*	stuck insect	apple cider vinegar or non	Geissmann, et al. [67]
camera	delta trap	*Lobesia botrana*	stuck insect	pheromone	Ünlü, et al. [50]
camera	Trapview	*Cydia pomonella*	stuck insect	five-component sex pheromone-kairomone blend	Preti, et al. [68], Preti, et al. [40]
camera	delta trap	*Cydia pomonella*	stuck insect	pheromone	Schrader, et al. [69]

References:

7. Mankin, R.; Machan, R.; Jones, R. In *Field testing of a prototype acoustic device for detection of Mediterranean fruit flies flying into a trap*, Proc. 7th Int. Symp. Fruit Flies of Economic Importance, **2006**; 2006; pp 10-15.
8. Potamitis, I.; Rigakis, I.; Vidakis, N.; Petousis, M.; Weber, M., Affordable bimodal optical sensors to spread the use of automated insect monitoring. *Journal of Sensors* **2018,** 1-25.
9. Frewin, A.; Lopez, B.; Cox, A.; Hoffman, E.; Hazell, J., Comparison of two traps for monitoring California red scale (Hemiptera: Diaspididae). *Florida Entomologist* **2019,** 102, (3), 586-591.
10. Rustia, D. J. A.; Lin, C. E.; Chung, J.-Y.; Zhuang, Y.-J.; Hsu, J.-C.; Lin, T.-T., Application of an image and environmental sensor network for automated greenhouse insect pest monitoring. *Journal of Asia-Pacific Entomology* **2020,** 23, (1), 17-28.
12. Roosjen, P. P.; Kellenberger, B.; Kooistra, L.; Green, D. R.; Fahrentrapp, J., Deep learning for automated detection of *Drosophila suzukii*: potential for UAV‐based monitoring. *Pest Management Science* **2020**.
13. Jiang, J.-A.; Tseng, C.-L.; Lu, F.-M.; Yang, E.-C.; Wu, Z.-S.; Chen, C.-P.; Lin, S.-H.; Lin, K.-C.; Liao, C.-S., A GSM-based remote wireless automatic monitoring system for field information: A case study for ecological monitoring of the oriental fruit fly, *Bactrocera dorsalis* (Hendel). *Computers and electronics in agriculture* **2008,** 62, (2), 243-259.
14. Holguin, G. A.; Lehman, B. L.; Hull, L. A.; Jones, V. P.; Park, J., Electronic traps for automated monitoring of insect populations. *IFAC Proceedings Volumes* **2010,** 43, (26), 49-54.
17. Kim, Y.; Jung, S.; Kim, Y.; Lee, Y., Real-time monitoring of oriental fruit moth, *Grapholita molesta*, populations using a remote sensing pheromone trap in apple orchards. *Journal of Asia-Pacific Entomology* **2011,** 14, (3), 259-262.
25. Jiang, J.-A.; Lin, T.-S.; Yang, E.-C.; Tseng, C.-L.; Chen, C.-P.; Yen, C.-W.; Zheng, X.-Y.; Liu, C.-Y.; Liu, R.-H.; Chen, Y.-F., Application of a web-based remote agro-ecological monitoring system for observing spatial distribution and dynamics of *Bactrocera dorsalis* in fruit orchards. *Precision Agriculture* **2013,** 14, (3), 323-342.
26. Shieh, J.-C.; Wang, J.-Y.; Lin, T.-S.; Lin, C.-H.; Yang, E.-C.; Tsai, Y.-J.; Tsai, H.-T.; Chiou, M.-T.; Lu, F.-M.; Jiang, J.-A., A GSM-based field monitoring system for *Spodoptera litura* (Fabricius). *Engineering in agriculture, environment and food* **2011,** 4, (3), 77-82.
40. Preti, M.; Favaro, R.; Knight, A. L.; Angeli, S., Remote monitoring of *Cydia pomonella* adults among an assemblage of nontargets in sex pheromone‐kairomone‐baited smart traps. *Pest management science* **2021b,** 77, (9), 4084-4090.
44. Pérez Aparicio, A.; Llorens Calveras, J.; Rosell Polo, J. R.; Martí, J.; Gemeno Marín, C., A cheap electronic sensor automated trap for monitoring the flight activity period of moths. *European Journal Of Entomology, 2021, vol. 118, p. 315-321* **2021**.
50. Ünlü, L.; Akdemir, B.; Ögür, E.; Şahin, İ., Remote monitoring of European grapevine moth, *Lobesia botrana* (Lepidoptera: Tortricidae) population using camera-based pheromone traps in vineyards. *Turkish Journal of Agriculture-Food Science and Technology* **2019,** 7, (4), 652-657.
51. Ascolese, R.; Gargiulo, S.; Pace, R.; Nappa, P.; Griffo, R.; Nugnes, F.; Bernardo, U., E‐traps: A valuable monitoring tool to be improved. *EPPO Bulletin* **2022,** 52, (1), 175-184.
52. Okuyama, T.; Yang, E.-C.; Chen, C.-P.; Lin, T.-S.; Chuang, C.-L.; Jiang, J.-A., Using automated monitoring systems to uncover pest population dynamics in agricultural fields. *Agricultural Systems* **2011,** 104, (9), 666-670.
53. Chuang, C.-L.; Jiang, J.-A., ICT-based Remote Agro-Ecological Monitoring System–A Case Study inTaiwan. *Journal of Communication, Navigation, Sensing and Services (CONASENSE)* **2014,** 1, (1), 67-92.
54. Goldshtein, E.; Cohen, Y.; Hetzroni, A.; Gazit, Y.; Timar, D.; Rosenfeld, L.; Grinshpon, Y.; Hoffman, A.; Mizrach, A., Development of an automatic monitoring trap for Mediterranean fruit fly (*Ceratitis capitata*) to optimize control applications frequency. *Computers and Electronics in Agriculture* **2017,** 139, 115-125.
55. Brydegaard, M.; Gebru, A.; Kirkeby, C.; Åkesson, S.; Smith, H. In *Daily evolution of the insect biomass spectrum in an agricultural landscape accessed with lidar*, EPJ Web of Conferences, 2016; EDP Sciences: 2016; p 22004.
56. Song, Z.; Zhang, B.; Feng, H.; Zhu, S.; Hu, L.; Brydegaard, M.; Li, Y.; Jansson, S.; Malmqvist, E.; Svanberg, K., Application of lidar remote sensing of insects in agricultural entomology on the Chinese scene. *Journal of Applied Entomology* **2020,** 144, (3), 161-169.
57. Jansson, S.; Brydegaard, M., Passive kHz lidar for the quantification of insect activity and dispersal. *Animal Biotelemetry* **2018,** 6, (1), 6.
58. Kirkeby, C.; Wellenreuther, M.; Brydegaard, M., Observations of movement dynamics of flying insects using high resolution lidar. *Scientific reports* **2016,** 6, 29083.
59. Tobin, P. C.; Klein, K. T.; Leonard, D. S., Gypsy moth (Lepidoptera: Lymantriidae) flight behavior and phenology based on field-deployed automated pheromone-baited traps. *Environmental entomology* **2009,** 38, (6), 1555-1562.
60. Hirafuji, M.; Yoichi, H.; Watanabe, T.; Asai, M.; Hu, H.; Tanaka, K.; Fukatsu, T.; Kiura, T.; Ninomiya, S.; Nagatsuka, T. In *Real-time insect monitoring system by using Field Server*, World conference on agricultural information and IT, IAALD AFITA WCCA, 2008; 2008; pp 277-282.

61. Solis-Sánchez, L. O.; Castañeda-Miranda, R.; García-Escalante, J. J.; Torres-Pacheco, I.; Guevara-González, R. G.; Castañeda-Miranda, C. L.; Alaniz-Lumbreras, P. D., Scale invariant feature approach for insect monitoring. *Computers and electronics in agriculture* **2011,** 75, (1), 92-99.
62. Doitsidis, L.; Fouskitakis, G. N.; Varikou, K. N.; Rigakis, I. I.; Chatzichristofis, S. A.; Papafilippaki, A. K.; Birouraki, A. E., Remote monitoring of the *Bactrocera oleae* (Gmelin)(Diptera: Tephritidae) population using an automated McPhail trap. *Computers and Electronics in Agriculture* **2017,** 137, 69-78.
63. Guarnieri, A.; Maini, S.; Molari, G.; Rondelli, V., Automatic trap for moth detection in integrated pest management. *Bulletin of Insectology* **2011,** 64, (2), 247-251.
64. Liu, B.; Hu, Z.; Zhao, Y.; Bai, Y.; Wang, Y., Recognition of Pyralidae Insects Using Intelligent Monitoring Autonomous Robot Vehicle in Natural Farm Scene. *arXiv preprint arXiv:1903.10827* **2019**.
65. Ding, W.; Taylor, G., Automatic moth detection from trap images for pest management. *Computers and Electronics in Agriculture* **2016,** 123, 17-28.
66. Sipos, K.; Madár, S.; Markó, M.; Pénzes, B., The possibility of automation of sex pheromone trapping: Tested on *Resseliella theobaldi* (Barnes)(Dip. *African Journal of Agricultural Research* **2012,** 7, (5), 1410-1413.
67. Geissmann, Q.; Abram, P. K.; Wu, D.; Haney, C. H.; Carrillo, J., Sticky Pi is a high-frequency smart trap that enables the study of insect circadian activity under natural conditions. *PLoS Biology* **2022,** 20, (7), e3001689.
68. Preti, M.; Moretti, C.; Scarton, G.; Giannotta, G.; Angeli, S., Developing a smart trap prototype equipped with camera for tortricid pests remote monitoring. *Bulletin of insectology* **2021a,** 74, (1), 147-160.
69. Schrader, M. J.; Smytheman, P.; Beers, E. H.; Khot, L. R., An open-source low-cost imaging system plug-in for pheromone traps aiding remote insect pest population monitoring in fruit crops. *Machines* **2022,** 10, (1), 52.
