# Supplementary material for: Automatic Detection of Moths (Lepidoptera) with a Funnel Trap Prototype
Source: Insects. 2023 Apr 13;14(4):381. doi: 10.3390/insects14040381 (PMC10145081; doi:10.3390/insects14040381)
Supplement: Supplementary file 1 [file insects-14-00381-s001.zip › Supplement_2.pdf]

Supplement S2. Technological description of the ZooLog VARL trap

Classical VARL trap in the CSALAMON pheromone trap family is based on a funnel (www.csalomoncsapdak.hu). When a flying insect attracted by the species-specific pheromone stick enters the funnel, it flies through the lower hole. The insect enters the lower container and cannot escape through that narrow opening. Although the probability of escape appeared low under natural field conditions, this value can be high enough to give a high bias between the number of detections and catches.

To avoid this bias, we developed a blow-off device that moves the insects downwards into the sampling container. Using this blow-off device, we significantly increased the catching rate, detection accuracy, and precision.

Structure of the trap:

The sensor case is integrated into the VARL trap and fastened by two screw anchors (Figure S1). To prevent pheromone contamination, the closure of the sensor case is ensured by stainless steel, the hat on the upper and lower part, and a glass tube in the detecting band. The stainless steel and glass materials provide the multi-pheromone usage of the sensor.

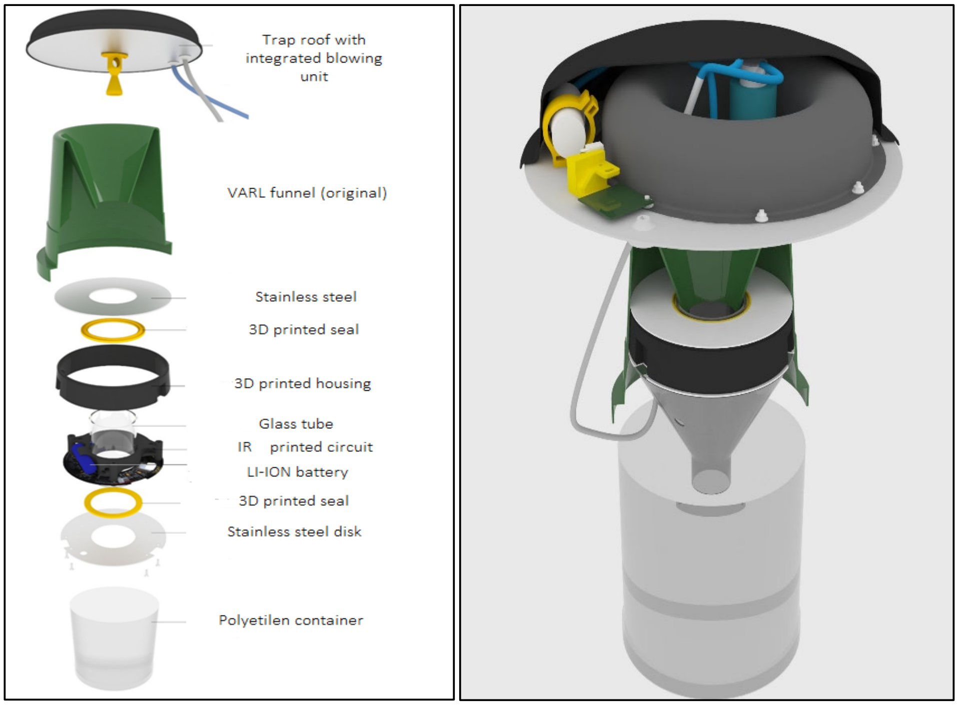

Figure S1. 3D interpretation of the construction of the modified VARL probe

Detection is done by the IR sensor, which has two precisely positioned infrared transmitters and 2x8 pieces of infrared sensors (directed toward the transmitter). Its effective detection area is 35 mm, which exceeds the size of the opening of the VARL trap (Figure S2).

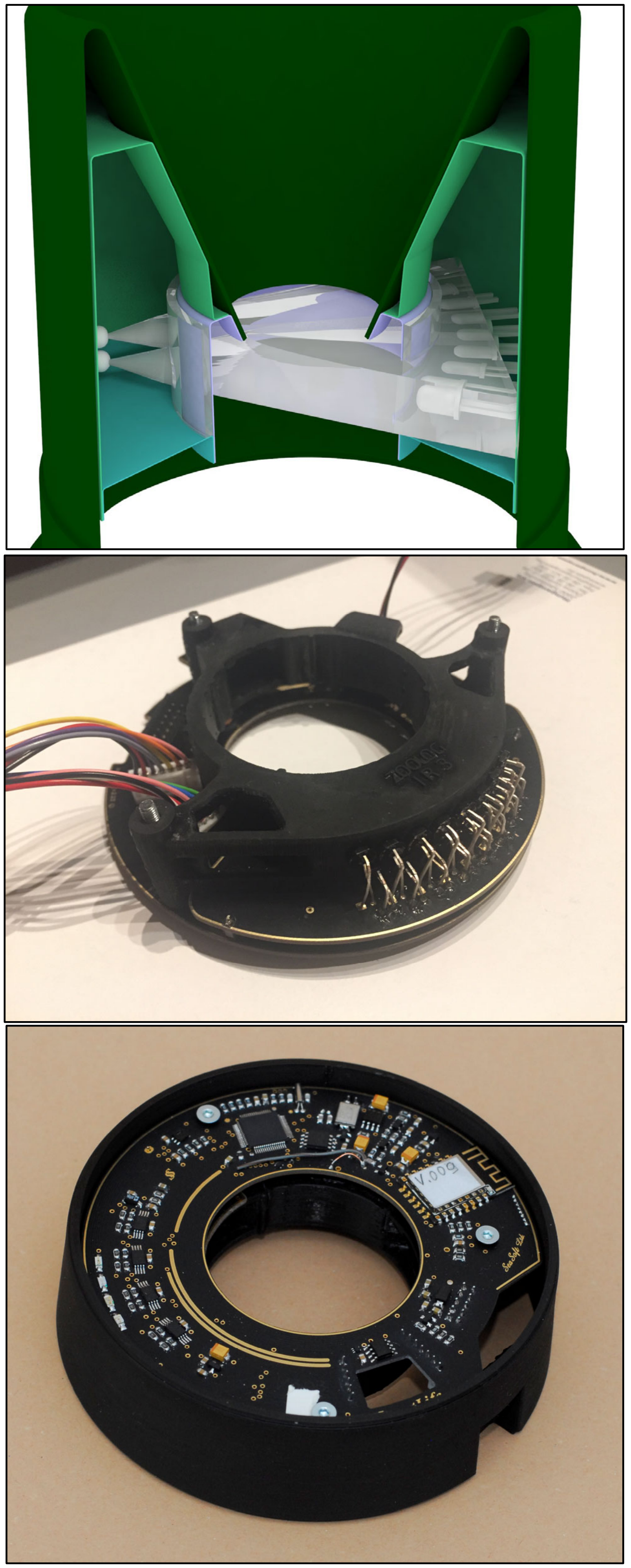

Figure S2. IR senor-ring built in the VARL trap

The glass design reaches under the probe using the original funnel system, thus preventing animals from escaping. During detection, the blow-off device (Figure S3) built into the trap roof is activated, which orients the detected animal to the collecting area with slight pressure (0.5 bar), ensuring precise detection. The trap roof is designed not to be an obstacle for the animals and provides air circulation similar to the original VARL trap. So the pheromone spread is the same as in the original VARL traps, thus comparing data gathered by electronic and standard VARL trap systems.

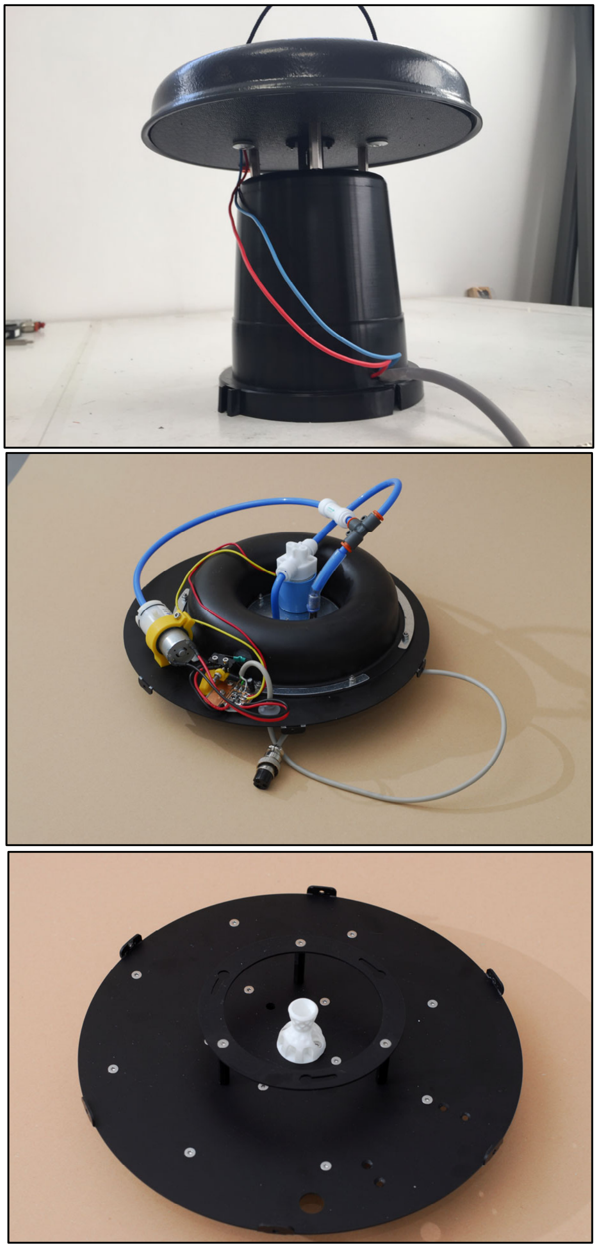

Figure S3. Construction of the modified VARL probe

An inflatable rubber ring serves as an air pressure tank for the blow-off system (Figure S3). The continuous constant pressure is inflated by a 12V mini pump (Figure S4/1). When the rubber ring reaches the optimal pressure, its rubber wall presses the analog switch (Figure S4/2), and

the mini pump stops working. When the pressure in the rubber ring is reduced, the analog switch is released, and the pump starts working again. This control system ensures that there is always adequate air pressure in the air tank.

The blow-off unit has a constant-pressure solenoid valve (Figure S4), which opens after detection and blows in response to a signal from the blow-off control unit (Figure S4).

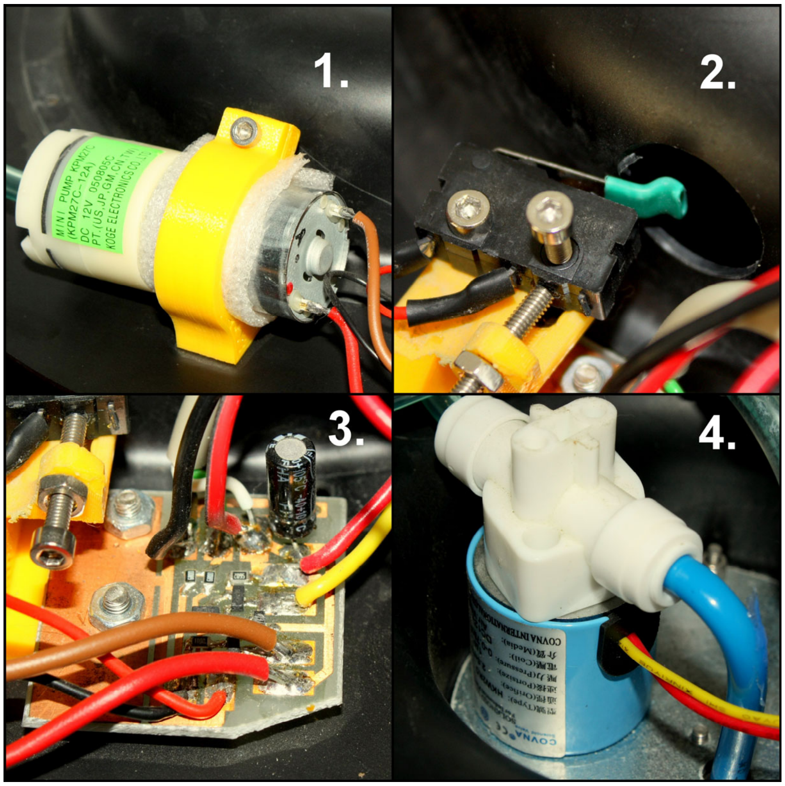

Figure S4. The electronic parts of the blow-off system**:** Air pump (1), analog switch (2), control unit (3), solenoid valve (4)

The integrated blow-off device receives a power supply and control from the probe electronics. Compressed air (see the blue pipe in Figure S4/4) gets to the cover plate from an outer container through a pressure-reducing regulator. Compressed air needed for the blow-off gets into the trap area through a LAVAL nozzle controlled by the magnetic valve built into the trap roof.

Production technology:

On the lower panel of the sensor, an IP67 waterproof ring seal provides the perfect closure of the communication power supply cable. The 12-core, shielded cable connects to the communication unit equipped with a solar cell power supply and GPRS module through an IP67 D-Sub connector.

The sensor case cannot be disassembled; it is made together by adhesion (Sika Flex) for compact design and prevents water and vapor from getting in. Before bonding, plastic surfaces must be treated with Sika Primer 206, and metal surfaces must be treated with Sika Aktivator.

During assembly, 3g silica gel is put inside the sensor case to absorb the vapor.
