# Supplementary material for: Automatic Detection of Moths (Lepidoptera) with a Funnel Trap Prototype
Source: Insects. 2023 Apr 13;14(4):381. doi: 10.3390/insects14040381 (PMC10145081; doi:10.3390/insects14040381)
Supplement: Supplementary file 1 [file insects-14-00381-s001.zip › Supplement_3.pdf]

Supplement S3. Initial phases of the development of the ZooLog VARL probe

After laboratory tests of the infrared sensor, it was inbuilt into VARL traps, which were developed for flying insects. With the new probes we conducted field tests at Elvira major close to Érd village (Pest County, Hungary) in August 2016 and between June and August 2017. We used the plum fruit moth *Grapholita funebrana* as the target species. During the two years of field tests, we conducted behavioral observations and recorded the attractiveness and effectiveness of the different traps from the beginning of the second and the third flying peak of the plum fruit moth.

This short description presents three types of initialization with which we tried to capture effectively moth species and minimalize disturbances, namely: trap equipped with top and down ventilator, compressed air.

Trap, equipped with a ventilator on the top

During the test in 2016, we tested probes equipped with a small ventilator (the ventilator takes place at the top of the trap in an open box). The upper part of the trap was black and the sample container was transparent. The ventilator blows down insects entering.

Following data filtering, compared with manual counting we were able to provide an exact specimen number about the population of actively flying plum fruit moths (Figure S1.).

There were constructional problems with this probe type which affected its usability. The ventilator took place in an open box that directly contacted the moth and dispersed the scales from the wings of the moth (and sometimes also the body of the animals), which adheres to the wall of the traps and attracted other animals, like wasps and bees. The latter increased the number of non-relevant catches.

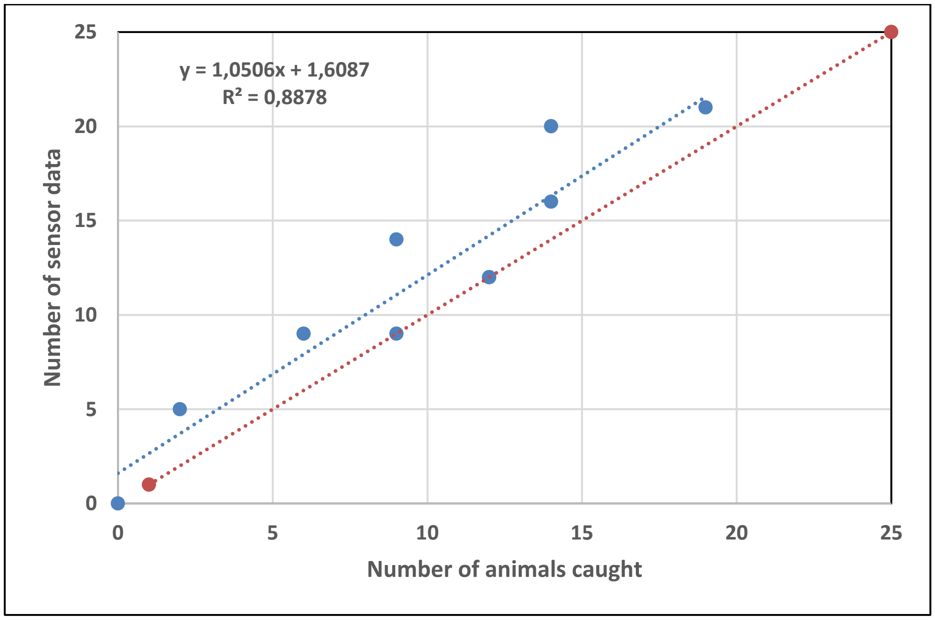

Figure S1. Correlation of the data provided by sensors and the real number of animals caught with VARL trap equipped with a ventilator at the top of the probe.

Trap, equipped with a ventilator on the bottom

In 2017 we modified the probe by replacing the ventilator from the top of the trap to the bottom into a box closed by a net (Figure S2.). The aim was to create a vacuum, which sucks in the insects. The field tests and observations showed that on many occasions the new ventilator apparatus begin to work without any detectable reason. In that way, we got far more signals than the real number of trapped animals. We have more explanation for this failure:

- The moth was able to stay stable on the glass tub covering the sensor, in spite of the vacuum effect of the working ventilator. The moth was able to move on the tube, which meant continuous detection of a single, same specimen.
- In case of successful trapping till it died, the moth was flittering in the upper part of the container, i.e. in front of the sensor. In that case, the sensor provided also signals continuously.

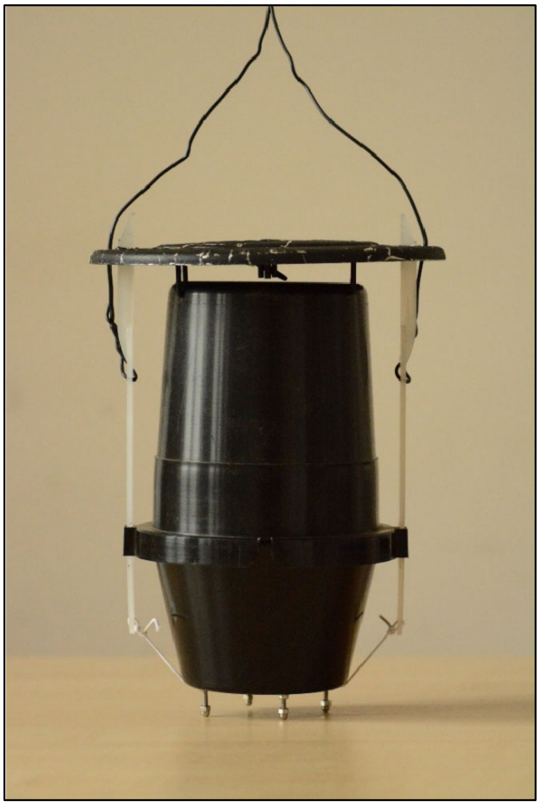

Figure S2. Trap with a ventilator at the bottom part.

In spite of different data filtering technics used we were unable to find a correlation between the data provided by sensors and observed data (Figure S3.).

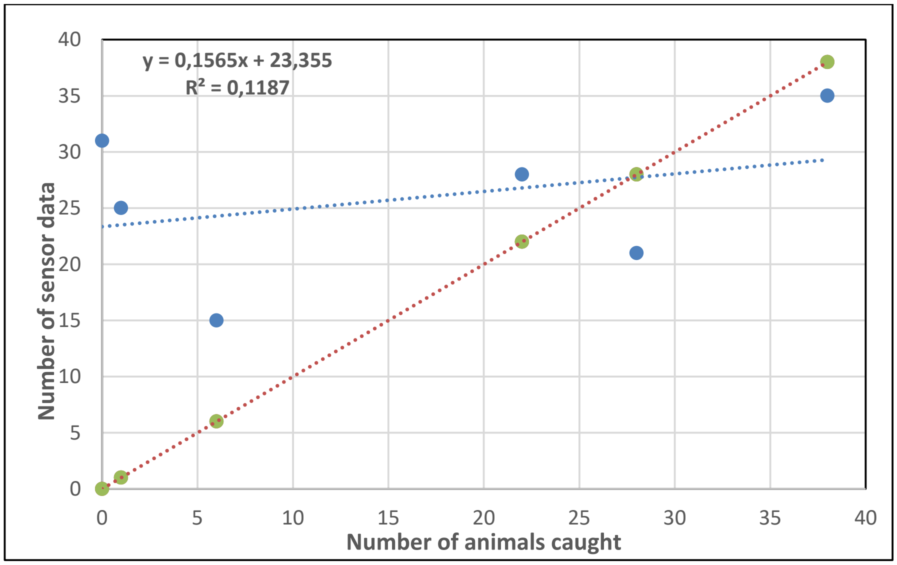

Figure S3. Correlation of the data provided by sensors and the real number of animals caught with VARL trap equipped with a ventilator at the bottom part.

Two control traps were placed beside the trap:

1. black upper part, transparent sample container, sensor;
2. white upper part, transparent sample container, without sensor.

Control probes showed that at the time of field tests population of active plum moths was far larger than the caught value with modified VARL traps.

At the VARL trap equipped with an automatic ventilator, fewer specimens caused far more false detection signals. Besides the too many signals, the probe had some constructional problems as well. The traps were unattractive for the moths, the animals did not fall into the trap and if it happened they easily found their way out of the trap. It could have several reasons:

- The black, dark, bottom of the trap was unattractive to the insects. Even if they fall into the traps searching for the source of the pheromone, they were flittering up towards the light (causing also false signals).
- The too-long glass tube which covers also the sensory part hindered the falling of the animals, which instead of moving down, flew out from the trap (Figure S4).

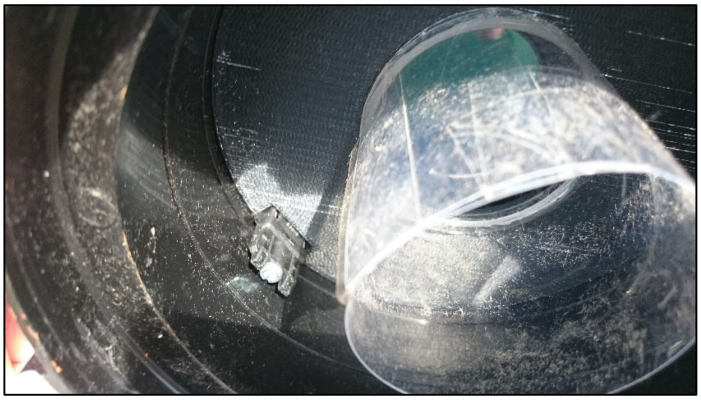

Figure S4. The glass tube in the trap, in front of the sensors.

- In case of a successful vacuum effect, because of the small size of the sample container, the moths easily found their way out of the trap. Another problem was, that because of the too-big ventilator box they are able to rest on it and climb out from the trap (Figure S5.).

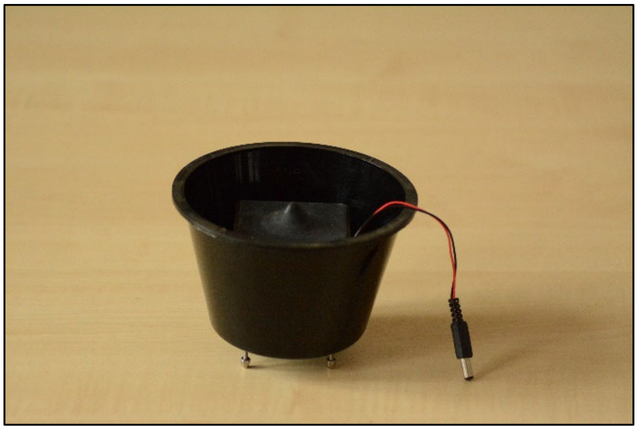

Figure S5. The closed ventilator box is located at the bottom of the trap.

According to the above-mentioned problems, we tested the probes with a different setup. We made the bottom of the trap transparent (because of the light sensitivity of the sensors we had to use a black (dark) upper part) (Figure S6.).

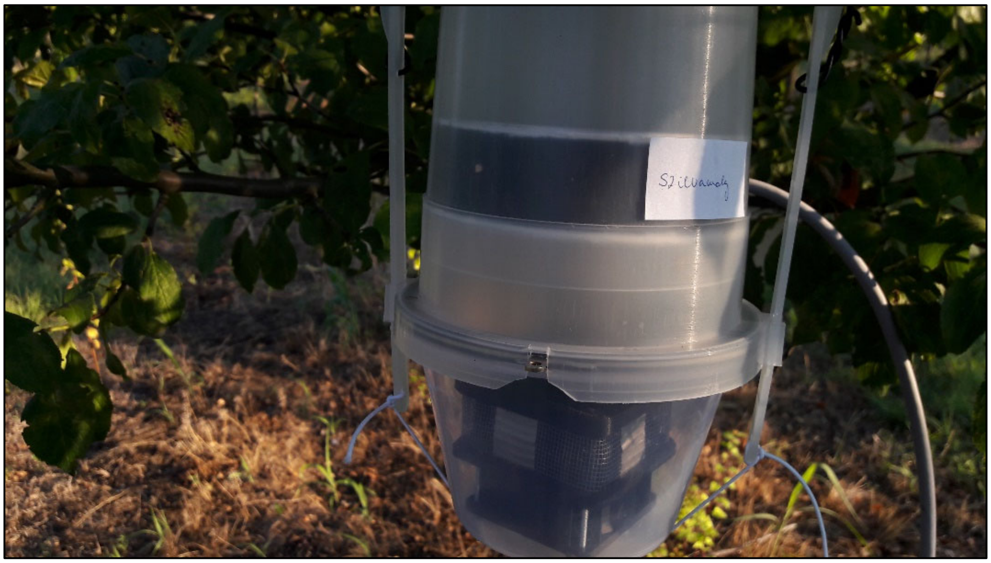

Figure S6. A VARL probe version with a transparent bottom part.

Conclusion deriving from the tests of the new version:

- The colour of the upper part (sensor house) and the long glass tube had no effect on the number of animals caught.

- The ventilator apparatus positively affected the escaping capacity of insects. (The moths sit on it and after that, they climb out easily.)
- The transparent sample container was more effective, because basically, animals move towards the light, in this case into the trap, which resulted in more effective trapping.

Traps working with compressed air (blowpipe)

The new type of VARL trap was modified. Because of the light sensitivity of the sensor, the upper part remained black (dark) and the bottom part was replaced by a transparent longer plastic tube. Because the version equipped with a ventilator was proven ineffective, we used another technic: blowpipe with compressed air. With this method, we were able to catch still actively the animals. One of the main advantages of this version was that the tank containing compressed air took place outside the probe (did not occupy the place and help the animals to escape). In case of animal arrived at the trap the sensor activated the blowpipe which blew the insect into the sample container (Figure S7.).

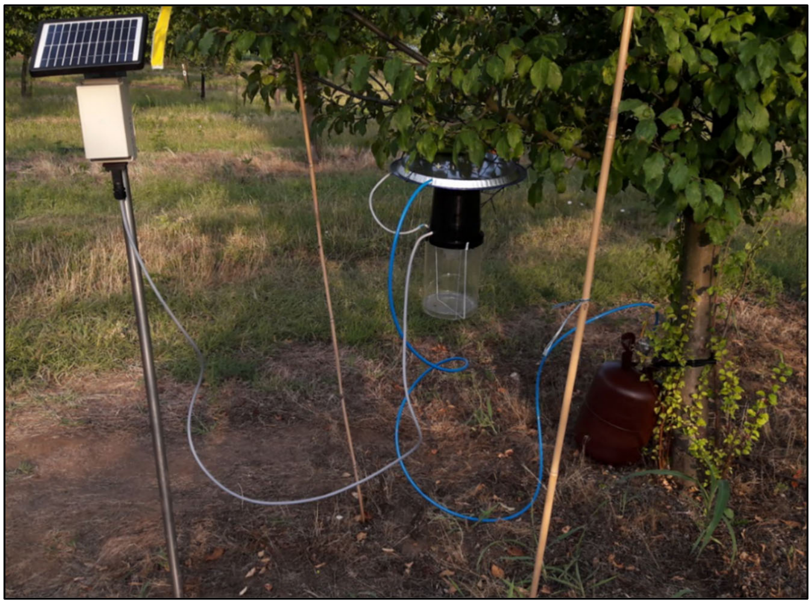

Figure S7. VARL trap working with compressed air**.**

Two control traps were placed beside the trap:

1. black upper part, transparent sample container, sensor;
2. white upper part, transparent sample container, without sensor.

The light sensitivity of the sensor remained a problem (i.e. many false signals). To solve this problem we stuck black tape at the upper 5 cm of the transparent sample container. This also hindered the escaping success of the animals (they were unable to find the way outside and rested in the dark part of the sample container) (Figure S8).

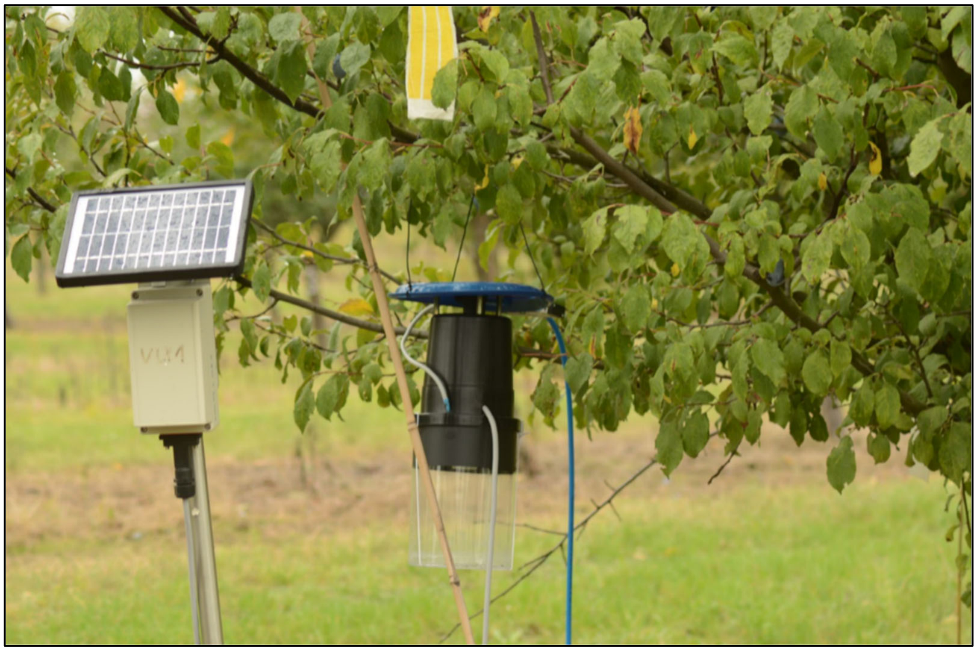

Figure S8. Trap with blowpipe and with black tape at the upper part of the sample container

Due to continuous monitoring, we were able to record which signal belonged to the actual capture of the trap. With a learning machine based on these observations, we were able to run more accurate data filtering. We were able to separate actual trapping from environmental noise (Table S1).

Table S1. Animal –non-animal sensor signals produced by VARL traps during field observations

2017.07.19. 4:30-7:10	Sensors								Moth	Not moth	Distribution
VL-06	1	2	3	4	5	6	7	8			
4:35:21	2	246	2296	203	9	12	4	4		x	
4:43:24	4	6	8	160	607	549	419	8	x		
4:43:25	0	3	7	0	39	4	766	5		x	
4:47:55	6	7	7	8	14	808	1629	1330	x		
4:49:30	511	511	7	7	13	13	13	10		x	
4:49:38	1023	236	48	5	7	9	9	9	x		
4:54:21	198	1438	176	9	14	9	6	9		x	
4:56:19	1029	1492	406	8	13	12	14	9	x		
5:00:29	3	3	4	4	7	7	255	255		x	
5:00:30	5	6	7	5	1023	23	12	13		x	
5:00:31	2	3	127	127	37	7	6	6		x	
5:00:33	3	3	6	7	8	10	12	337		x	
5:00:34	4	4	5	5	20	60	105	255		x	
5:03:54	7	11	19	8	21	9	3	222		x	
5:03:55	1	4	0	11	0	16	19	579		x	
5:04:04	5	4	10	12	22	28	7	500		x	
5:05:20	3	5	10	566	873	353	5	7		x	
5:06:22	4	3	5	5	9	7	97	511		x	
5:15:06	6	9	15	88	280	315	511	301	x		
5:15:12	0	1	0	0	2	295	5	1		x	
5:16:44	2	5	5	6	26	1481	1380	138	x		
5:19:58	44	628	853	441	24	12	9	8	x		
5:23:17	13	591	943	671	86	12	6	3	x		
5:46:19	511	186	23	21	26	22	21	16			
5:46:20	2	70	256	3	2	7	0	1			
5:48:13	103	160	338	852	813	262	48	21			
5:48:25	289	511	511	511	126	41	37	24			
5:48:30	4	5	19	255	255	255	13	11			
5:48:31	2	2	2	5	12	27	389	27			
5:49:23	4	23	511	491	298	20	12	9		x	
5:49:45	19	23	43	39	215	387	447	164		x	
5:51:16	137	255	255	200	29	22	20	11		x	
5:51:17	6	8	77	255	255	255	255	185			
5:52:35	255	35	12	4	7	9	8	5		x	
5:52:36	346	0	1	5	9	15	7	0		x	
5:52:37	511	247	61	14	7	11	14	11		x	
5:52:47	63	63	34	8	10	7	9	10		x	
5:52:49	322	14	46	37	53	21	20	18		x	
5:53:13	4	4	5	14	51	292	389	449			
5:54:18	41	46	51	42	31	18	5	576			
5:59:56	317	103	32	11	2	8	12	4		x	
6:01:54	37	54	259	430	549	369	90	34			
6:01:58	27	36	44	44	45	56	255	255			
6:06:29	50	72	213	644	931	528	35	30	x		
6:06:46	17	22	26	35	543	860	838	355	x		
6:08:07	4	5	8	34	130	511	511	246	x		
6:13:22	255	255	255	255	147	115	31	17	x		
6:14:16	101	163	409	771	511	96	52	27	x		
6:20:24	110	392	451	175	143	100	261	254	x		

Compared to the control traps, versions with blowpipe (compressed air) caught a bit lower amount of animals, but there were no differences in the magnitude of the number of specimens observed using of previous versions.

In the case of traps equipped with a blowpipe, the actual number of caught insects could be correlated with the manually filtered data provided by the sensors (Figure S9.). This type of trap seems to be accurate. However, there was a need to decrease the size of the tank of compressed air to make setting and transporting the traps easier.

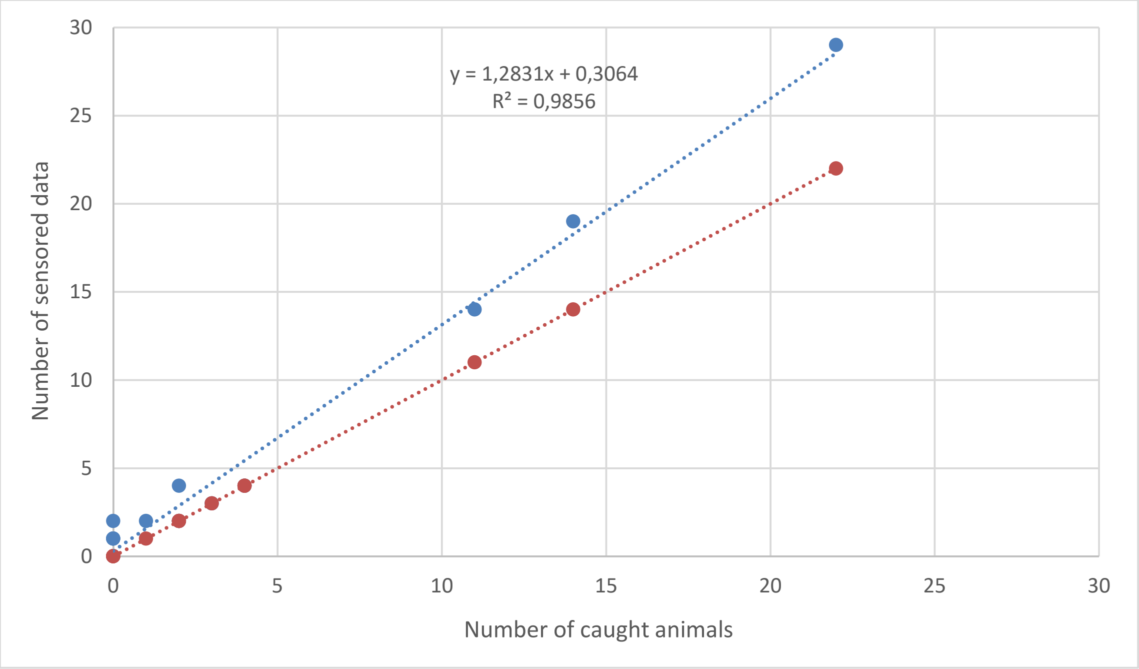

Figure S9. Correlation of the data provided by sensors and the real number of animals caught with VARL trap equipped with a blowpipe.
