# Supplementary material for: Automatic Detection of Moths (Lepidoptera) with a Funnel Trap Prototype
Source: Insects. 2023 Apr 13;14(4):381. doi: 10.3390/insects14040381 (PMC10145081; doi:10.3390/insects14040381)

Supplement S4. Automatic detection of individuals for the five target species over time during the monitoring period. The separate figures present data gained from different probes. Local polynomial regression fitting (loess) was applied for smoothing time-series data.

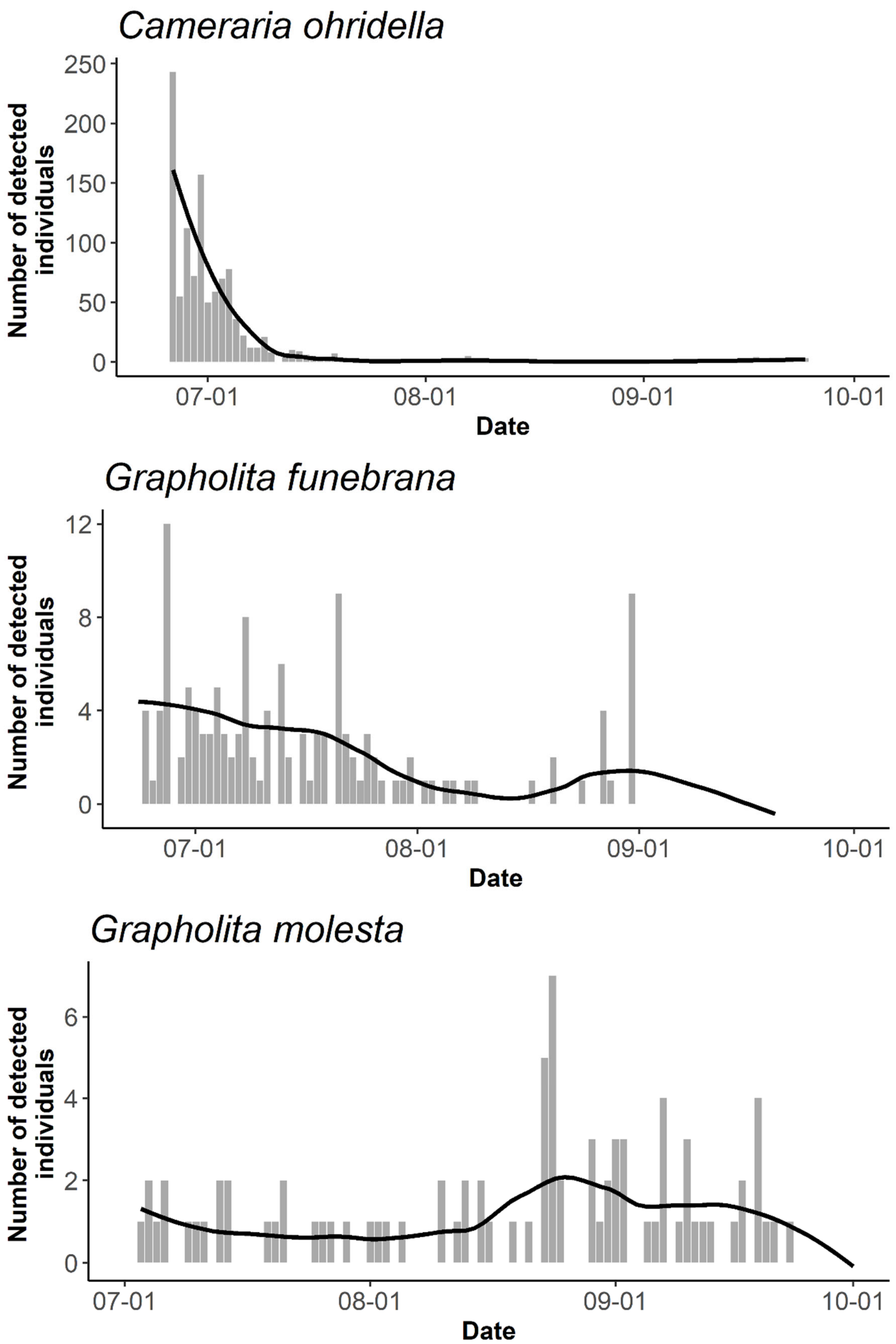

Cameraria ohridella
250
200
150
100
50
0
Number of detected individuals
07-01
08-01
09-01
10-01
Date
Grapholita funebrana
12
8
4
0
Number of detected individuals
07-01
08-01
09-01
10-01
Date
Grapholita molesta
6
4
2
0
Number of detected individuals
07-01
08-01
09-01
10-01
Date

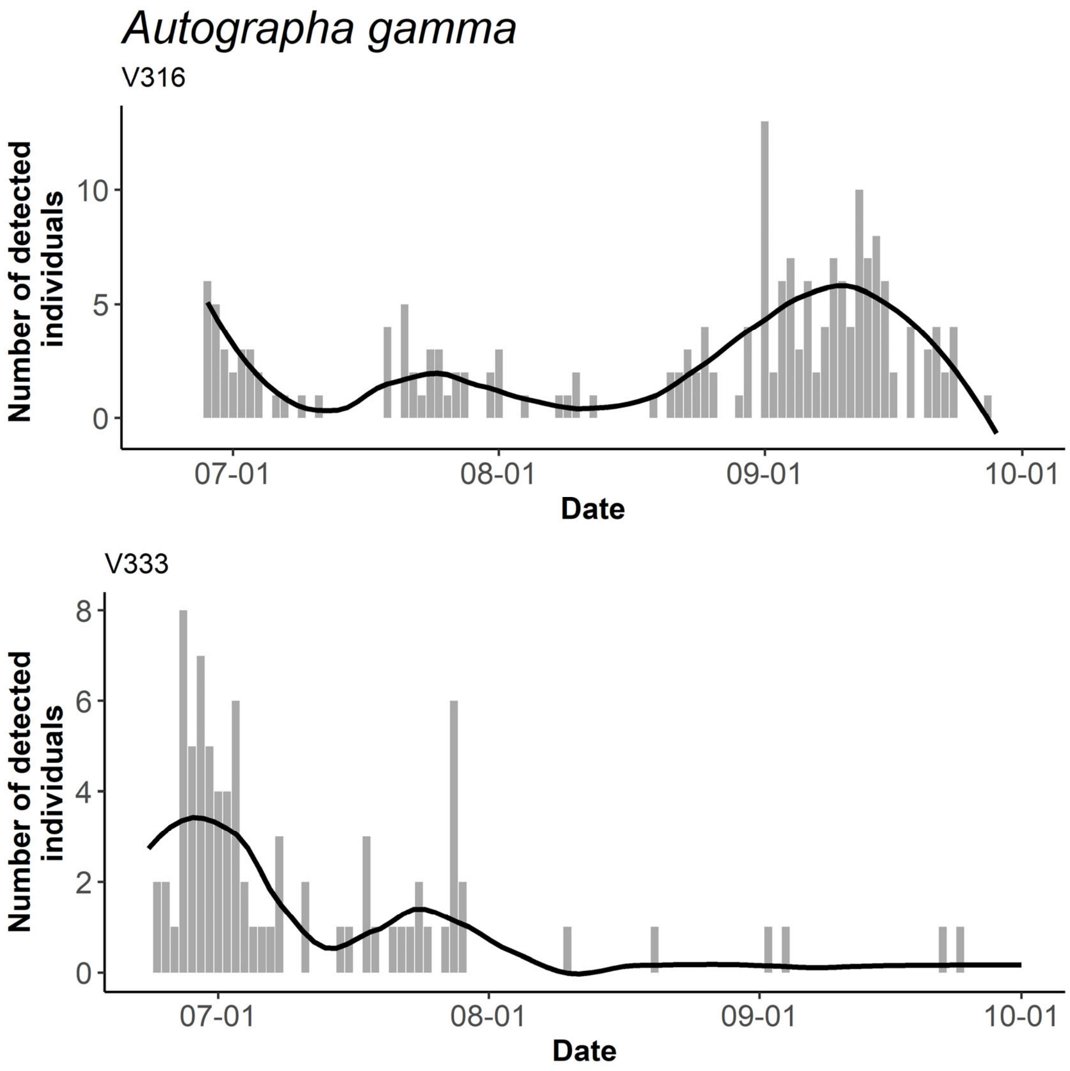
Autographa gamma
V316
Number of detected individuals
0
5
10
07-01
08-01
09-01
10-01
Date
V333
Number of detected individuals
0
2
4
6
8
07-01
08-01
09-01
10-01
Date

Agrotis segetum

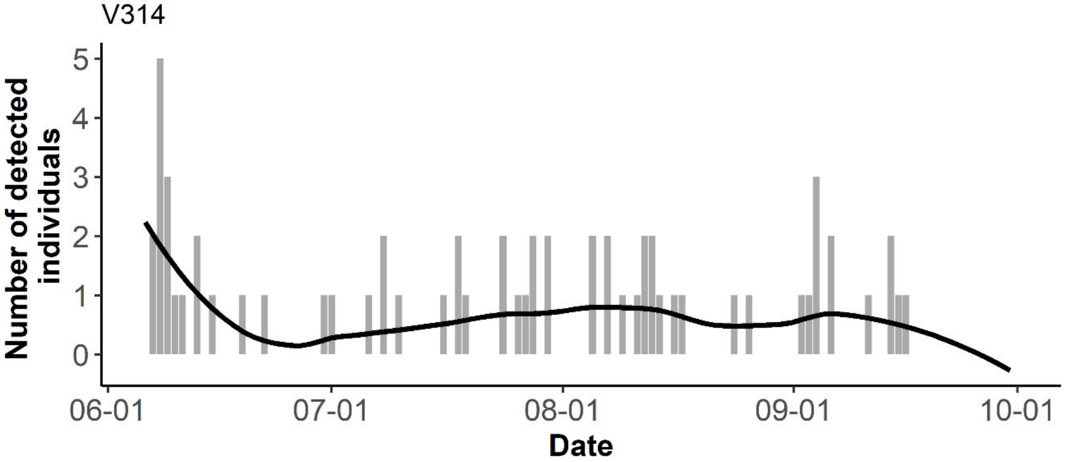

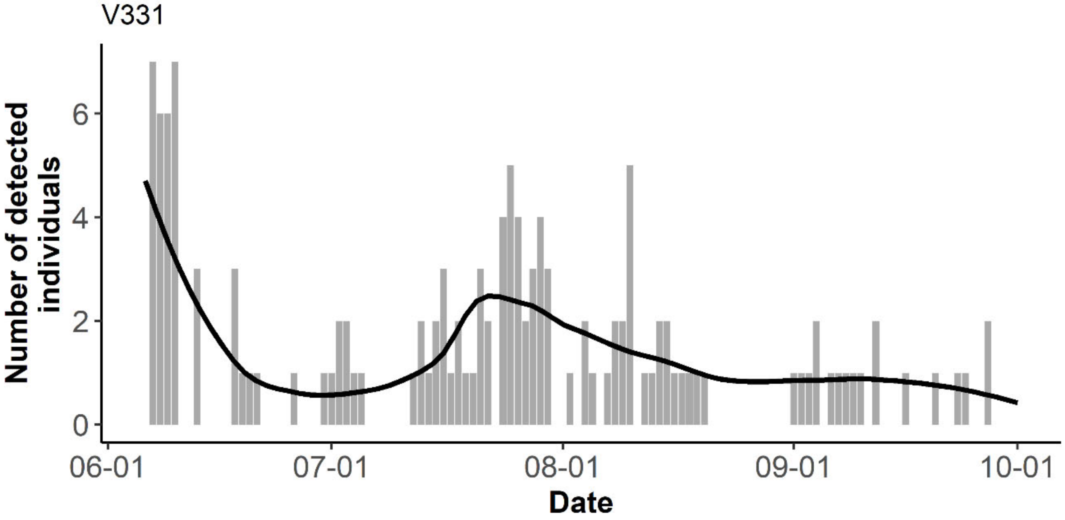

Supplement: Supplementary file 1 [file insects-14-00381-s001.zip › Supplement_4.pdf]
